# Tumour cells can escape antiproliferative pressure by interferon-β through immunoediting of interferon receptor expression

**DOI:** 10.1186/s12935-023-03150-y

**Published:** 2023-12-08

**Authors:** Felix Hiebinger, Aiste Kudulyte, Huanting Chi, Sebastian Burbano De Lara, Doroteja Ilic, Barbara Helm, Hendrik Welsch, Viet Loan Dao Thi, Ursula Klingmüller, Marco Binder

**Affiliations:** 1 Research Group “Dynamics of Early Viral Infection and the Innate Antiviral Response”, Division Virus-Associated Carcinogenesis (F170), German Cancer Research Center (DKFZ), Heidelberg, Germany; 2 Faculty of Biosciences, Heidelberg University, Heidelberg, Germany; 3 Schaller Research Group, Department of Infectious Diseases, Virology, University Hospital Heidelberg, Heidelberg, Germany; 4 German Centre for Infection Research (DZIF), Partner Site Heidelberg, Heidelberg, Germany; 5 Division of Systems Biology of Signal Transduction (B200), German Cancer Research Center (DKFZ), Heidelberg, Germany; 6 German Center for Lung Research (DZL) and Translational Lung Research Center Heidelberg (TRLC), Heidelberg, Germany; 7 Medical Faculty Heidelberg, Heidelberg University, Heidelberg, Germany

**Keywords:** Interferon, Immunoediting, Cell proliferation, Signalling, Interferon resistance, Cancer, Hepatocellular carcinoma

## Abstract

Type I interferons (IFNs) play a central role not only in innate immunity against viral infection, but also in the antitumour response, e.g. through a direct impact on cell proliferation. Particularly for cancer arising in the context of chronic inflammation, constant exposure to IFNs may constitute a strong selective pressure during tumour evolution. Expansion of neoplastic subclones resistant to the antiproliferative effects of IFNs may contribute to immunoediting of tumours, leading to more aggressive disease. Experimental evidence for this development of IFN-insensitivity has been scarce and its molecular mechanism is unclear. In this study we demonstrate that six weeks exposure of cells to IFN-β in vitro reduces their sensitivity to its antiproliferative effects, and that this phenotype was stable for up to four weeks. Furthermore, we observed substantial differences in cellular sensitivity to growth inhibition by IFN-β in a panel of ten different liver cancer cell lines, most prominently in a pair of highly dedifferentiated cell lines, and least in cells from well-differentiated tumours. In both, long-term IFN selection and in dedifferentiated tumour cell lines, we found IFNAR2 expression to be substantially reduced, suggesting the receptor complex to be a sensitive target amenable to immunoediting. Beyond new insights into possible molecular processes in tumour evolution, these findings might prove valuable for the development of biomarkers allowing to stratify tumours for their sensitivity to IFN treatment in the context of patient tailored therapies.

**Supplementary Information:**

The online version contains supplementary material available at 10.1186/s12935-023-03150-y.

## Introduction

Interferons (IFNs) were first reported by Isaacs and Lindenmann as an effective antiviral substance [1]. Type I IFNs (e.g. IFN-α and -β) are the major constituents of the cell-intrinsic antiviral defense, rapidly produced and secreted upon virus recognition by innate immune sensors. They signal in an auto- or paracrine manner through the IFN alpha receptor (IFNAR), a heterodimeric receptor composed of a low-(IFNAR1) and a high-affinity (IFNAR2) receptor chain. Upon IFNAR1/IFNAR2/IFN-trimerisation, receptor associated kinases (TYK2, JAK1) phosphorylate the transcription factors signal transducer and activator of transcription (STAT) 1 and 2, that further associate with IRF9 to the trimeric interferon stimulated gene factor (ISGF)3. After translocating to the nucleus, ISGF3 induces the expression of hundreds of interferon-stimulated genes (ISGs) that in concert put cells into a highly antiviral state [2].

Beside their central role in antiviral innate immune defence, type I IFNs have long been described as strong antitumour agents [3–5]. This activity can either be indirect by affecting angiogenesis [6–10] or by attracting and activating immune cells [11–14], or direct by reducing proliferation of tumour cells and sensitising them for apoptosis [3, 15–17]. Impeding proliferation is complex and cell-type dependent [18], but generally involves the inhibition of cyclin-dependent kinases (CDKs) [15, 17, 19–23]. Interestingly, while many antiviral ISGs are expressed robustly even after very transient IFNAR engagement (“robust” ISGs), antiproliferative effects require tight binding of IFN to the receptor and long-lasting trimerisation of the receptor complex (“tuneable” ISGs) [24]. Therefore, IFN-β, having the highest affinity to both receptor subunits, exhibits the strongest antiproliferative potency among the type I IFNs [16, 25–27]. The cytostatic or even cytotoxic effects of type I IFNs have led to the early proposing of IFNs as therapeutics in oncology [3]. However, more recent research found a complex interplay of IFNs in oncogenesis and tumour progression, including pro-oncogenic effects [28–30], and there is increasing evidence that beyond viral infection also oncogenic events, such as DNA damage, chromosomal instability or micronuclei formation, lead to the induction of type I IFN [31–33]. Whereas this initially might be part of the physiological defence strategy against malign transformation, it could be exploited and drive tumour progression once overcome by a mutating cancer cell. Tumours comprise a competitive environment of proliferating cells with high genetic and epigenetic heterogeneity [34–36]. This enables individual tumour cells (subclones) capable of evading extrinsic selective pressures to outcompete less successful subclones and quickly become dominant in the course of tumour evolution. The immune system may be the strongest and best-studied extrinsic pressure, e.g. through adaptive responses to tumour-specific neoantigens. The immune-directed evolution leading to the emergence and dominance of specific tumour subclones capable of escaping this immune response is called immunoediting of the tumour. This is a serious complication in immunotherapy approaches using chimeric antigen receptor (CAR) T-cells, as immunoediting can deplete the tumour of the targeted neoantigen, hence, evading therapeutic T-cell responses [37].

A cancer entity frequently associated with IFN is hepatocellular carcinoma (HCC). Typically, it arises in the context of chronic inflammation, with hepatitis B (HBV) and hepatitis C (HCV) virus causing 33% and 21% of all primary liver cancers [38]. Particularly for HCV, significant and chronically ongoing intrahepatic production of IFN and consequent ISG induction have been reported [39–41]. Moreover, also in the context of HBV, which does not induce such a strong IFN-signature, and in the context of non-viral aetiologies, such as alcohol abuse or metabolic syndrome, long-lasting inflammation (hepatitis) likely goes along with prolonged cytokine and IFN exposure [42]. Although chronic inflammation is well-known to promote the development of cancer [43], we hypothesise that an IFN-rich milieu primarily constitutes a hostile environment for malignly transformed cells due to the antiproliferative activity of IFN. Hence, it is feasible that continuous IFN presence at the site of tumourigenesis acts as a strong selection pressure and may be a major contributor to the immunoediting of the tumour, eventually leading to development and outgrowth of tumour subclones resistant to the antiproliferative effect of type I IFN. This concept is supported by numerous studies and observations across a range of tumour entities, and has previously been comprehensively reviewed [44]. Still, experimental investigations addressing the process have been limited so far.

In the present study, we aimed to confirm and measure the proliferative impact of type I IFN onto dividing cells. In vitro*,* we could demonstrate the selective advantage of IFN-insensitivity in a defined genetic model (IFNAR-deficient cells) as well as in long-term high-dose IFN-β treatment of cells. In the latter case, we observed development of significant insensitivity towards the antiproliferative activity of IFN, which was relatively stable even weeks after withdrawal of IFN, reminiscent of IFN-driven immunoediting in a proliferating tumour environment. We then analysed ten different liver cancer cell lines based on the hypothesis that more advanced, potentially more aggressive, tumours would have undergone longer selection and, hence, might give rise to highly resistant phenotypes. Indeed, among advanced, poorly differentiated tumours we identified two lines that were virtually completely resistant to the antiproliferative effects of IFN-β. This phenotype was not due to a general lack of IFN-signalling but was selective for the cytostatic effect. The only significant molecular difference we could identify was a substantial downregulation of the IFNAR2 receptor chain in the lowly sensitive cells. Intriguingly, this downregulation was also apparent in our in vitro selection experiment, suggesting IFNAR, especially IFNAR2, to be a sensitive target for cells to modulate their susceptibility to antiproliferative effects of type I IFN.

## Materials and methods

### Materials and cell lines

IFN-β (8499-IF-010) was purchased from R&D Systems. Antibodies anti-calnexin (rabbit, polyclonal, Enzo Life science: ADI-SPA-865-F), anti-STAT1 (mouse, monoclonal, BD: 610,115), anti-STAT2 (mouse, monoclonal, Santa Cruz: sc-514-193), anti-STAT1-pY701 (mouse, monoclonal, BD: 612,232) and anti-STAT2-pY690 (rabbit, monoclonal, Cell Signaling: D3P2P) were used and detected with anti-mouse IgG (goat, polyclonal, Sigma-Aldrich: A4416-5X1ML) or anti-rabbit IgG (goat, polyclonal, Sigma-Aldrich: A6154-5X1ML).

Oligonucleotide primers for qPCR were ordered from Sigma-Aldrich and were based on the following sequences (5’–3’):

GAPDH fwd/rev: TCGGAGTCAACGGATTTGGT/TTCCCGTTCTCAGCCTTGAC, IFIT1 fwd/rev: GAATAGCCAGATCTCAGAGGAGC/CCATTTGTACTCATGGTTGCTGT, IFNAR1 fwd/rev: CTCCGCGTACAAGCATCTGA/TGGAGGAAGTAGGAAAGCTTGTATT, IFNAR2 fwd/rev: TGCGAAATTTCCGGTCCATC/ACCTTCAAATCTTCTGGTTTACTCA, IRF1 fwd/rev: CCTGACTCCAGCACTGTCG/TGGGTGACACCTGGAAGTTG, USP18 fwd/rev: ATCCGGAATGCTGTGGATGG/AGATATGCAGTTTCCTGCCAGT, IFNB1 fwd/rev: CGCCGCATTGACCATCTA/GACATTAGCCAGGAGGTTCTC, eGFP fwd/rev: CTACCCCGACCACATGAAGC/AAGAAGATGGTGCGCTCCTG.

Human liver cancer cell lines were obtained from JCRB Cell Bank (Osaka, Japan; HLE, HLF, HuH1, HuH6, HuH7), LGC Standards (Wesel, Germany; PLC/PRF/5, SNU182, SNU387, HepG2) and DSMZ (Braunschweig, Germany; Hep3B). A549 alveolar carcinoma and HEK293T human embryonic kidney cell line were available within DKFZ, Heidelberg and non-neoplastic, SV40 large T antigen-immortalised hepatocyte cells PH5CH [45] were kindly provided by Prof. Volker Lohmann, Heidelberg. Human pluripotent stem cell line WA09 (Wicell) were purchased from ATCC. Combined IFNAR1 and IFNLR1 knockout (KO) cell lines were generated using the lentiCRISPR v2 system and published previously [46].

### Cell culture

Cells were grown in high glucose *Dulbecco’s modified Eagle’s medium* (DMEM, Thermo Fisher Scientific) supplemented with 10% fetal calf serum (FCS, Thermo Fisher Scientific), 1% non-essential amino acids (Thermo Fisher Scientific) and 1% penicillin–streptomycin (Thermo Fisher Scientific) at 37 °C, 85% relative humidity, and a CO_2_ saturation of 5%. In accordance with ATCC’s recommendations, SNU182, SNU387 and HepG2 cells were cultured in *Rosewell Park Memorial Institute 1640* medium (RPMI 1640, Thermo Fisher Scientific) supplemented the same as DMEM. Twice a week, cells were detached with 0.05% trypsin (Thermo Fisher Scientific) and passaged 1:2 to 1:20. For long-term storage, cells were suspended in FCS supplemented with 10% DMSO and stored in liquid nitrogen. The stem cell line WA09 were cultured in mTeSR1 medium (STEMCELL Technologies) on matrigel (Corning) coated plates and differentiated to mature hepatocyte-like-cells as previously described [47]. Cells were checked for mycoplasma contamination and authenticated by *short tandem repeat* (STR) analysis (DSMZ, Braunschweig, Germany). Cell counting was performed using a *CellDrop BF* automated cell counter (DeNovix).

### Lentiviral pseudoparticle production and transduction

Transgene expressing cell lines were generated by lentiviral transduction. To produce lentiviral pseudoparticles, HEK293T cells were transfected with pCMV-dr8.91, pMD2.G (Didier Trono, EPFL, Lausanne, Switzerland) and the lentiviral vector of interest (pWPI_EF1a_Neo_H2B-mCherry, pWPI_EF1a_Blr_H2B-mCherry, pWPI_EF1a_Blr_eGFP-nHA) at a 3:1:3 ratio via the polyethylemine (PEI, Polysciences) method. In brief, 1 × 10^6^ cells were seeded on a 6 cm dish, and the next day medium was replaced with penicillin–streptomycin-free DMEM one hour before transfection. Then, 45 µl PEI and 455 µl OptiMEM (Thermo Fisher Scientific) were mixed with 500 µl OptiMEM containing a total of 15 µg of plasmid DNA. After vortexing and incubating for 30 s, the mix was added dropwise to the dish. After 6 h, the medium was replaced with fresh complete DMEM. Supernatant containing lentiviral particles (LVP) was harvested after 48 h of incubation at 37 °C, sterile filtered (0.2 µm pore size, GE Healthcare) and stored at − 80 °C. 2 × 10^4^ target cells / well were seeded on a 24-well plate and the next day, medium was replaced with 500 µl / well of LVP-containing supernatant. After 24 h, it was exchanged with fresh medium containing the appropriate selection antibiotic (5 µg/ml blasticidin, MP Biomedicals; 500 µg/ml geneticin (G418), Santa Cruz Biotechnology). In A549, PH5CH and HepG2, G418 was used at 1000 µg/ml.

### Fluorescence microscopy and flow cytometry

Fluorescence microscopic images were taken on a *Nikon Eclipse Ti* and processed with *ImageJ* software (National Institutes of Health). Cell quantification was performed by flow cytometry. Cells were detached with 0.05% trypsin and resuspended in 100 µl phosphate buffered saline (PBS, Applichem) supplemented with 1% FCS. Cellular lumps were dispersed by pipetting the suspension through a cell-strainer sieve (Corning). The number of cells expressing mCherry or enhanced green fluorescent protein (eGFP) was counted on a LSRFortessa flow cytometer with *BD FACSDiva* software in the RFP and FITC channel, respectively. Population sizes were determined with *FlowJo* software.

### RNA quantification by RT-qPCR

RNA was transcribed reversely into cDNA and quantitative PCR (qPCR) was performed. First, RNA was isolated with the *Monarch RNA miniprep kit* (New England Biolabs) according to the manufacturer’s protocol. Briefly, 1–2 × 10^5^ cells were lysed in 300 µl buffer and after several washing and centrifugation steps, eluted in 50 µl nuclease-free water. Second, the concentration of RNA was determined with a NanoDrop spectrophotometer and reverse transcription was carried out with the *High-Capacity cDNA Reverse Transcription Kit* (Thermo Fisher Scientific) according to the manufacturer’s protocol. Third, cDNA was diluted 1:20 in ddH2O and 6 µl of the product were mixed with 9 µl *iTaq Universal SYBR Green Supermix* (Bio-Rad Laboratories) containing both forward and reverse primers for the gene of interest at a final concentration of 0.25 µM. qPCR was done in triplicates in hard-shell 96-well PCR plates in a *CFX96 Real-Time PCR* detection system (Bio-Rad Laboratories, programme: 3 min at 95 °C, then 44 cycles of 10 s at 95 °C, 30 s at 60 °C, fluorescence emission measurement). To enable comparison of mock levels across different cell lines, mRNA abundance of target genes was calculated relatively only to GAPDH, not normalising to a reference sample (2^−ΔCT^ instead of 2^−ΔΔCT^) [48]. Induction kinetics were assessed by normalising ΔC_T_-values of the gene of interest to its maximum expression within each cell line for each replicate separately. For a quantitative comparison of maximum induction levels, new cDNA was prepared from all peak samples and RNA levels were adjusted throughout the three replicates to 83.4 ng per setup. After performing RT-qPCR, results were displayed as 2^30−Ct^ typically per 4.2 ng total RNA. The same analysis was performed for the target genes in Additional file 3: Fig. S3b and Additional file 5: Fig. S5a–d.

### Protein quantification by immunoblotting

For protein quantification, SDS–polyacrylamide gel electrophoresis (PAGE) was performed with whole cell lysates followed by immunoblotting (western blot). After washing with PBS, 1–2 × 10^5^ cells were lysed in 80 µl *RIPA* buffer (Cell Signaling Technology) supplemented with 10 mM NaF as phosphatase inhibitor and 10% protease inhibitor cocktail (Pierce Protease Inhibitor Tablets EDTA-Free, Thermo Fisher Scientific) by mechanical scratching and vigorous up-and-down-pipetting of the samples. Next, the lysates were centrifuged for 10 min at 16,000 rcf and the supernatants transferred to other microtubes. The whole procedure was performed at 4 °C. Protein concentration was determined using the *Pierce BCA kit* (Thermo Fisher Scientific) using microplates. Samples were then mixed with 6 × protein sample buffer (97,5 mM TRIS (pH 6.8), 30% glycerol, 3% SDS, 7,5% β-mercaptoethanol, 0.05% bromophenol blue) and equal amounts of protein (5–10 µg) were loaded onto SDS–polyacrylamide gels (8% acrylamide:bisacrylamide (29:1), 0.1% TEMED, 0.1% saturated ammonium persulfate solution, 375 mM TRIS (pH 8.8), 192 mM glycine, 0.1% SDS) and separated at 120 V in 1 × TRIS–glycine-sulfate buffer (TGS: 25 mM TRIS (pH 8.3), 192 mM glycine, 0.1% SDS) in a *Tetra Vertical Electrophoresis Cell* (Bio-Rad Laboratories). Proteins were blotted onto a methanol activated PVDF membrane (pore size: 0.2 µm, Bio-Rad Laboratories) by wet transfer at 4 °C and 350 mA for 2:15 h in blotting buffer (25 mM TRIS (pH 8.3), 150 mM glycine, 20% methanol). Membranes were blocked at room temperature in 5% milk in PBS-T (PBS supplemented with 0.1% Tween-20, AppliChem) and after 3 washing steps incubated over night at 4 °C on a shaker with the primary antibody diluted 1:1000 in 5% bovine serum albumin (BSA, Sigma-Aldrich) in PBS-T. Phospho-antibodies were dissolved in 5% BSA in TBS-T (20 mM TRIS (pH 7.6), 150 mM NaCl, 0.1% Tween-20), instead. The next day, three washing steps (5–10 min) were performed in PBS-T/TBS-T before incubating the membrane with the secondary antibody diluted 1:10,000 (mouse) or 1:20,000 (rabbit) in 5% BSA/PBS-T. After three final washing steps, 300 µl of electrochemiluminescence substrate (Thermo Fisher Scientific) were added and membranes were imaged using the ECL ChemoCam 3.2 instrument (INTAS Science Imaging Instruments). Raw densitometry values were quantified with *ImageJ* and normalised to the cumulated signal of all samples within one experiment. Hence, “densitometry values” refers to the percentage of signal received from one sample in comparison to all other samples. For pSTAT quantification, the densitometry value of each pSTAT measurement was normalised to the mean expression of the corresponding STAT in the corresponding cell line and experiment.

### Live-cell imaging

Live-cell imaging was performed using an *IncuCyte SX5* live-cell analysis system (Sartorius, software: IncuCyte 2020B) or an *IncuCyte S3* (Software: IncuCyte 2019B), respectively. For each experiment and condition, 1–4 images were taken from 3 separate wells (technical replicates).

### Growth assay

**Fig. 1:**
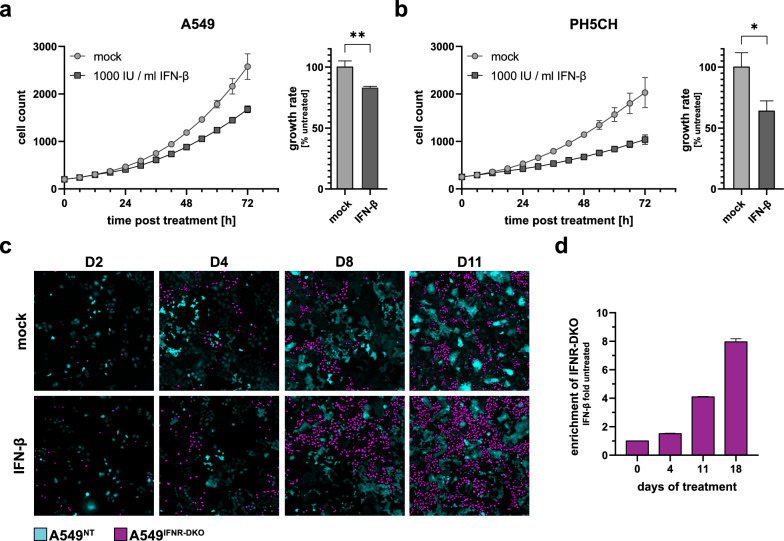
Antiproliferative effect of IFN-β on different cell lines. **a** A549 lung adenocarcinoma cells, or **b** PH5CH non-neoplastic liver cells were kept under 1000 IU/ml IFN-β or mock treated. Cell proliferation was monitored by automated life-cell imaging (IncuCyte S3). Data represents mean ± SD of three biologically independent repetitions. Unpaired student`s t-test. **c + d** A549^IFNR−DKO^ (H2B-mCherry labelled) and A549^NT^ control cells (cytosolic GFP labelled) were cocultured (initial ratio of 1:9) for 18 days in medium containing 5000 IU/ml IFN-β (or mock) and frequently split. **c** Representative microscopy images, more in Additional file 1: Fig. S1. **d** Ratio of A549^INFR−DKO^ to A549^NT^ cells was determined at days 0, 4, 11 and 18 by flow cytometry and normalised to mock condition. Data shows mean ± SD of two separately passaged cocultures

Depending on the cell line, 2–6 × 10^3^ of H2B-mCherry transgene-expressing cells were seeded onto a 96-well plate and stimulated the next day. Thereafter, plates were sealed with gas permeable film (Azenta Lifesciences) and put into an IncuCyte instrument for life-cell imaging (see above). Every 6 h, images were taken from 4 independent areas per well at 10 × magnification. Proliferation was monitored by automatic counting of mCherry-stained nuclei using the IncuCyte software (see above). To offset variance in the initial seeding number, data in Fig. 1a, b was normalised by dividing each measurement at 0 h by the initial cell count in mock of replicate 1 and multiplying subsequent counts by its reciprocal. Later, the exponential growth data (cell count) was transformed logarithmically and plotted in *PRISM 9.3* (GraphPad). The growth rate was determined by fitting a linear regression from the onset of growth inhibition to the general slowdown of proliferation due to overgrowth, typically between 24–72 h. IC_50_ values were determined in *PRISM* using the *[Inhibitor] vs. response (three parameters)* model for characterisation of the hepatoma cell panel and the four-parameter model for the characterisation of long-term selected cells, respectively.

### Migration assay

Cell migration was determined via scratch wound assays. Cells were seeded at 1–4 × 10^4^ per well on 96-well *ImageLock* microplates (Sartorius) coated with collagen I (Serva Electrophoresis). 16 h later, cells had reached 95% confluence and were treated with 5 µg/ml mitomycin c (Santa Cruz Biotechnology) for two hours to inhibit proliferation; the concentration was verified to efficiently block proliferation of all tested cell lines. Finally, scratching was performed with a wound maker tool (Sartorius) and after washing twice with PBS, fresh medium was added. Scanning was scheduled once per hour and cell numbers (1 image / well) monitored to ensure effective inhibition of proliferation. Wound confluence was determined as the proportion of cell-covered area within the original scratch wound. Next, migration rates were calculated as slopes of the linear regression (Δwound confluence [%]/h) ranging from 0 to 24 h. Since HuH6 exhibited a very irregular scratching pattern and tended to detach completely, 6 instead of 3 technical replicates were performed from which 3 were selected for each analysis based on the integrity of their wound margins.

The migration assay for human induced stem cell-derived hepatocyte-like-cells (HLCs) followed the procedure described below for invasion with minor modifications. Fully matured HLCs were generated as described in Dao Thi et al. [47] and dissociated by Accutase (Innovative cell technologies) and Y-27632 (STEMCELL Technologies) treatment. 2.5 × 10^4^ HLCs were seeded on an uncoated chamber of a 24-transwell in 500 µl hepatocyte basal medium (Lonza), with the lower compartment filled with hepatocyte culture medium (Lonza) supplemented with oncostatin-M (R&D system). The total cell number was assessed through Hoechst 33,342 (1:1000, Thermo Fisher Scientific) staining after fixation. Images were taken on Nikon ECLIPSE Ts2 fluorescence microscope (Software: NIS-Elements D) and then counted by CellProfiler [49].

### Invasion assay

Invasive potential was determined with a transwell system. 1 × 10^5^ cells were resuspended in 500 µl FCS-free medium and seeded on *matrigel*-coated chambers (24-well, 8.0-micron, Corning) or uncoated control inserts (Corning), respectively, after rehydrating them for 2 h in FCS-free medium. For fast-migrating cell lines HLE, HLF, SNU387 and PH5CH, 2.5 × 10^4^ cells per well were seeded instead. The lower chamber was filled with medium containing 10% FCS, establishing a chemoattractant gradient. After 24 h, cells that had not migrated, but remained on the upper surface were removed with a moist cotton swab and those that penetrated to the lower surface were fixed in 4% paraformaldehyde and stained with 1% crystal violet solution (20% ethanol). After air drying the chambers, whole well images were taken on a *Cell Observer* microscope (Software: ZEN 3.0 blue edition, Carl Zeiss) and total cell numbers were counted. Cut-off for evaluating invasiveness was set to > 1000 cells on the uncoated membrane, representing 1% of seeded cells. Invasiveness was calculated as ratio of migrated cells on a coated relative to a corresponding uncoated well.

### HBsAg measurement

Absolute concentrations of secreted HBsAg from the undiluted supernatants of HuH1, Hep3B, SNU182, SNU387 and PLC cells were detected using the HBsAg quantitative system (Architect, Abbott). Values > 0.05 IU/ml were considered positive.

### Viral infection

For the infection assay, 3.5 × 10^5^ cells were infected with 2% FCS DMEM / RPMI medium containing the attenuated recombinant vesicular stomatitis virus VSV*MQ [50] at an MOI of 10 (virus was kindly provided by Dr. Gert Zimmer, Institute of Virology and Immunoprophylaxis, Mittelhäusern, Switzerland). After six hours, medium was replaced with the fresh complete medium and after additional eighteen hours cells were harvested for qRT-PCR analysis.

### Sample preparation for mass spectrometry

For the full proteome characterisation, cells were lysed in buffer containing 25 mM Tris pH 7.6, 150 mM NaCl, 0.1% SDS, 1% NP-40, 1% Deoxycholic acid NA-salt, 1 mM Na_3_VO_4_, 10 mM NaF, 1 µg/mL Aprotinin, 0.1 mg/mL 4-(2-Aminoethyl) benzene sulfonyl fluoride-hydrochloride, 250 U/mL Benzonase, 10 U/mL DNase and PhosSTOP (4,906,845,001 Roche: 1 tablet/10 ml buffer). The total cell lysates were then incubated and rotated for 30 min at 4 °C. Samples were centrifuged for 15 min at 14,000 rpm at 4 °C, and the supernatants were transferred to new vials. The BCA Assay (Pierce) was used to estimate protein concentration, and 10 µg of total protein was used per sample for further processing. Protein digestion and clean-up were performed using an adapted version of the automated paramagnetic bead-based single-pot, solid-phase-enhanced sample-preparation (Auto-SP3) protocol [51] on a Bravo liquid handling platform (Agilent). Initially, protein disulfide bonds were reduced with 10 mM TCEP and alkylated with 40 mM CAA for 5 min at 95 °C. For bead preparation, Sera-Mag Speed Beads A and B (GE Healthcare) were vortexed until the pellet was dissolved. The suspension was placed on a magnetic rack, and the supernatant was removed after one minute. The beads were taken off the magnetic rack and suspended in water. This procedure was repeated three times. A total of 20 µL of bead A was combined with 20 µL of bead B, and the final volume was corrected to 100 µL with mass spectrometry-comptible-H_2_O (MS-H_2_O). A total of 5 µL of A + B beads mixture was added to each sample. The Bravo liquid handling platform (Agilent) was operated using the "Auto-SP3" protocol provided by Agilent. 100 mM TEAB buffer containing trypsin (enzyme/protein ratio of 1:25) was used for protein digestion, and samples were incubated overnight at 37 °C. After digestion, the recovered peptides were dried by vacuum centrifugation (1300 rpm at 45 °C), and stored at − 80 °C until use.

### Liquid chromatography–mass spectrometry (LC–MS/MS) analysis

For nano-flow LC–MS/MS analysis, an Ultimate 3000 HPLC (Thermo Fisher Scientific) was coupled to an Orbitrap Exploris 480 mass spectrometer (Thermo Fisher Scientific). Tryptic peptides were dissolved in 15 µl loading buffer (0.1% formic acid (FA), 2% ACN in MS-compatible H_2_O), and 3 µL were injected for each analysis. The samples were loaded onto a pre-column (PEPMAP 100 °C 18 5 µm 0.3 × 5 mm, Thermo Scientific) using a loading pump at a higher flow rate. After 4 min, a valve was switched, and peptides were delivered to an analytical column (75 µm × 30 cm, packed in-house with Reprosil-Pur 120 C18-AQ, 1.9 µm resin, Dr. Maisch) at a flow rate of 5 µL/min in 98% buffer A (0.1% FA in MS-H_2_O). After loading, peptides were separated using a 141 min gradient from 8 to 38% of buffer B (0.1% FA, 80% ACN in MS-H_2_O) at a 300 nL/min flow rate. The Orbitrap Exploris 480 mass spectrometer was operated in in data-independent mode (DIA), with an m/z range of 350–1400. Full scan spectra were acquired in the Orbitrap at 120,000 resolution after accumulation to the set target value of 300% (100% = 10^6^) and maximum injection time of 45 ms. DIA scans followed the full scans. Forty-seven isolation windows were defined, with an m/z range of 406–986. Spectra were generated in the orbitrap (isolation window 1 m/z) after fragmentation using higher energy collisional dissociation (HCD) at normalised collision energy (N)CE of 28% and acquired at 30,000 resolution after accumulation to the set target value of 1000% (100% = 10^5^) and maximum injection time of 54 ms.

### Database search, analysis, and availability of the mass spectrometry data

All DIA raw data files were analysed with a direct DIA workflow using Spectronaut 17.6 (Biognosys, Zurich, Switzerland). The Uniprot Homo sapiens reference proteome database was used for the Pulsar search. The default settings for database match include full specificity trypsin digestion, peptide length of between 7 and 52 amino acids, and maximum missed cleavage of 2. N-terminal methionine was removed during preprocessing of the protein database. Carbamidomethylation at cysteine was used as a fixed modification, and protein N-terminal acetylation and methionine oxidation were set as variable modifications. The false discovery rates (FDRs) were set as 0.01 for the peptide-spectrum match (PSM), peptide, and protein identification. For quantification, identified (Qvalue) was set for precursor filtering and MS2 quantification with the area as quantity type. The original mass spectrometric raw files Spectronaut files are available on the proteomeXchange PRIDE platform [52] http://www.proteomexchange.org, under the accession number PXD043761.

### Statistical analysis

For the statistical analysis of the proteomics data, a modified version of the MSPypeline, a python-based pipeline for full proteome analysis [53], was used. Briefly, for the generation of the scatter plot (outlier detection and comparison plots), an experiment comparison on level 0 was performed using log_2_-transformed raw intensities. For the generation of the volcano plot (statistical inference plots) log_2_-transformed raw intensities and a comparison on level 0 were used. Average fold changes between the cell lines were calculated, and statistical significance was assessed using the limma R package [54], which contains functionality specifically designed to handle high-dimensional biological data, implemented in MSPypeline.

All other statistical analysis was performed in PRISM 9.3 (Graphpad). For comparison of two conditions, unpaired t test was performed. For more than two conditions, ordinary one-way ANOVA was followed by Tukey’s test to correct for multiple comparisons. Appropriateness of the model was verified with Brown-Forsythe test to exclude unequal variance. ns = not significant, *p < 0.05, **p < 0.01, ***p < 0.001 and ****p < 0.0001.

## Results

### IFN-β inhibits growth of PH5CH and A549 cells.

Among type I IFNs, IFN-β reportedly exerts the strongest antiproliferative effect due to its high receptor affinity [16, 25–27]. To quantify antiproliferative effects with high temporal resolution, we monitored cell growth in a live-cell imaging approach on the IncuCyte platform. Both A549 alveolar carcinoma as well as immortalised non-neoplastic PH5CH hepatocytic cells [45] exhibited typical exponential growth. Confirming the antiproliferative effect of IFN-β, both cell lines tested grew significantly slower when treated with 1000 IU/ml of IFN-β (Fig. 1a, b left panels). We found the growth rate of A549 cells to be reduced to 83% upon IFN-β treatment, and to even 63% for PH5CH cells (Fig. 1a, b right panels).

We hypothesized that this severe proliferative disadvantage of cells exposed to IFN-β may constitute a substantial selective pressure in quickly proliferating tissue such as malignancies, and, hence, shape the evolution of tumours. To simulate this evolutionary effect in vitro, we cocultured wildtype A549 cells (labelled by cytosolic GFP expression) with “IFN-blind” A549 cells, lacking type I and III IFN receptors (IFNAR1/IRNLR1 double KO, DKO) (labelled by nuclear mCherry). By continuous exposure to 5000 IU/ml IFN-β and frequent passaging, we exposed the cells to a strong selective pressure. After 18 days of passaging, the fraction of IFN-insensitive cells increased eightfold in the IFN-treated culture as compared to the fraction in cocultures kept in IFN-free medium (Fig. 1c, d). This confirmed our primary assumption that reduced sensitivity to IFN-β could constitute a proliferative advantage in an IFN-rich microenvironment.

### Selection of IFN-β-resistant hepatocytes by long-term passaging

**Fig. 2:**
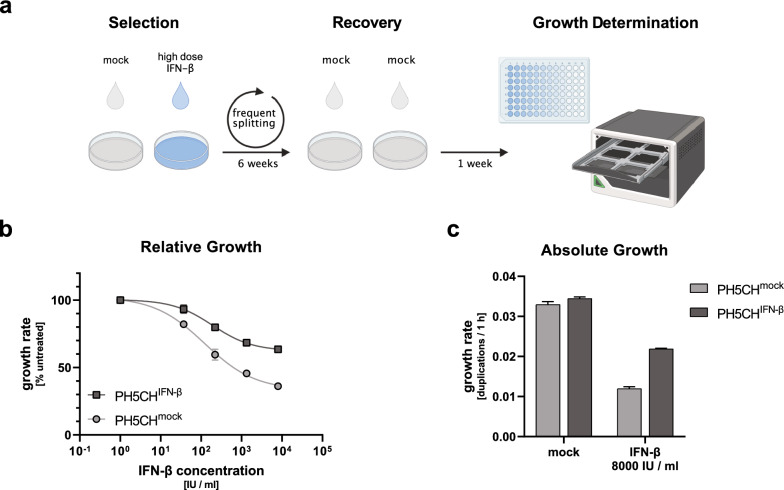
In vitro selection of IFN-β-insensitive cell populations upon long-term treatment. **a** Schematic overview over selection process. **b** After long-term (6 weeks) IFN-β treatment and IFN-free recovery (1 week), cells were stimulated with IFN-β and growth rates were determined relative to mock-treated control (included for simplicity at x = 10^0^). **c** Absolute growth rates (duplications per hour) of PH5CH^IFN−β^ and PH5CH^mock^ unstimulated and stimulated with 8000 IU/ml IFN-β. PH5CH^IFN−β^ corresponds to three independently passaged long-term selected cell populations, PH5CH^mock^ to three technical replicates of one population. Data displayed as mean ± SD

Within a population of cells, the response to IFN is heterogeneous and a fraction of cells remains unresponsive [55]. However, there is not much known about the molecular underpinnings of this effect, in particular it is unclear whether it is limited to random transcriptional noise [55] or whether there is a contribution of more stable effects, such as epigenetics. In the latter case, continuous exposure to IFN may favour the selection of cells with reduced sensitivity to IFN-mediated growth inhibition and may therefore contribute to oncogenic processes. To investigate this possibility, we subjected a population of non-malignant PH5CH cells to stringent selection pressure by IFN-β and frequent passaging for six weeks (Fig. 2a). To control for passaging related effects, one well was cultured in the absence of IFN. After 6 weeks of selection, cells were kept for 1 week in IFN-free medium to re-equilibrate in order to omit effects based on random transcriptional noise or transient upregulation of feedback regulators of IFN signalling. We then assessed growth rates of the cells in the absence or presence of different doses of IFN-β. Cells that underwent six weeks of IFN-pressure (PH5CH^IFN−β^) were substantially less affected by IFN-β-treatment than mock-selected cells (PH5CH^mock^), indicating successful selection of a less IFN-sensitive population (Fig. 2b, see Additional file 2: Fig. S2a for shorter selection periods). At the highest IFN dose PH5CH^IFN−β^ proliferated almost twice as fast (0.022 vs 0.012 h^−1^) as their PH5CH^mock^ counterparts (Fig. 2c). Notably, the same effect was present in each of the three populations that independently underwent six weeks of IFN-selection (Additional file 2: Fig. S2b, c). Moreover, we observed a slight increase of the basal proliferation for PH5CH^IFN−β^ over PH5CH^mock^ cells. Although this effect was small, it occurred in all three populations (Additional file 2: Fig. S2c). We further assessed the stability of the observed phenotype of decreased IFN-sensitivity after three more weeks of normal, IFN-free cultivation. Indeed, PH5CH^IFN−β^ maintained their relative IFN-resistance compared to PH5CH^mock^ albeit to a somewhat reduced extent (Additional file 2: Fig. S2d, e). In general, the reduced IFN-sensitivity was less characterised by a shift of the dose–response (IC_50_) but rather by an overall reduced growth inhibition across all concentrations, including the highest ones (10,000 IU/ml) (Fig. 2b, Additional file 2: Fig. S2d, b). Importantly, PH5CH^mock^ behaved very comparable to a freshly thawed aliquot of PH5CH cells that have not undergone the 6-week selection process, underscoring that indeed the presence of IFN constituted the major selective pressure (Additional file 2: Fig. S2d, e).

Taken together, our results indicate that even among a genetically homogeneous population of cells stable phenotypes exist that mediate increased resistance against the growth inhibitory effects of IFN signalling. Such cells exhibit a proliferative advantage when IFN is continuously present in the medium, and hence can be enriched by selection.

### Characterisation of a panel of ten hepatoma cell lines

Although tumour cells exhibit dysregulated proliferation, it is known that in many cases they remain susceptible to the growth inhibitory effects of IFN [16, 56, 57]. Particularly for tumours developing in the context of chronic inflammation, e.g., in chronic HCV infection, continuous presence of IFNs in the tumour microenvironment [39] may present a dominant selective pressure. We and others [44] therefore hypothesise that many advanced tumours have undergone selection to overcome the cytostatic effects of IFNs.

In order to test this hypothesis, we assessed a panel of rather well-characterised tumour cell lines regarding their growth response to IFN-β. We chose liver-derived cancer lines because of their high likelihood to have developed in the context of chronic virus infection. Eight stemmed from hepatocellular carcinoma (HCC) and two from juvenile hepatoblastoma [58] (Table 1). As a control, we used PH5CH cells, as they reportedly are non-neoplastic and have been immortalised in vitro. Previous HBV infection can be detected in some of the cell lines by presence of viral DNA integrates [59, 60], and in HuH1 and PLC we detected ongoing HBsAg secretion (Table 1, Additional file 3: Fig. S3a). HCV was unknown at the time of establishment of most of the cell lines and due to the lack of DNA integration cannot be detected retrospectively. Despite presence of viral components in some lines, there was no basal production of IFN-β in any of them (Additional file 3: Fig. S3b).

**Table 1:** Characteristics of studied cell lines

Cell line	Sex	Age	Primary tissue	Viral status	Cluster	Original publication (additional studies):	Year
PH5CH	M	58	Non-neoplastic hepatocytes	anti-HCV antibody	–	Noguchi and Hirohashi [45]	1996
PLC	M	24	Hepatoma	HBV DNA integrates HBsAg secretion	Epithelial-like	Alexander et al. [61] ([62–64])	1976
HuH1	M	53	Hepatoma	HBV DNA integrates HBsAg secretion	Epithelial-like	Huh et al.[62] ([63, 64])	1981
HuH6	M	1	Hepatoblastoma	None	Epithelial-like	Doi et al. [65] ([63, 64])	1976
HuH7	M	57	Well-differentiated HCC	None	Epithelial-like	Nakabayashi et al.[66] ([63, 64, 67, 68])	1982
HepG2	M	15	Hepatoblastoma	None	Epithelial-like	Aden et al.[69] ([63, 64, 67, 68])	1979
Hep3B	M	8	Differentiated HCC	HBV DNA integrates No HBsAg secretion	Epithelial-like	Aden et al.[69] ([63, 64, 67, 68])	1979
HLE	M	68	Undifferentiated HCC	None	Fibroblast-like	Doi et al.[70] ([63, 64, 68])	1975
HLF	M	68	Undifferentiated HCC	None	Fibroblast-like	Doi et al.[70] ([63, 64, 68])	1975
SNU182	M	24	HCC, grade III/IV	HBV DNA integrates No HBsAg secretion	Fibroblast-like	Park et al. [71] ([62–64])	1995
SNU387	F	41	HCC, grade III-IV/IV	HBV DNA integrates No HBsAg secretion	Fibroblast-like	Park et al. [71] ([62–64])	1995

*Cluster definition based on Fukuyama *et al*. *[63]

### Assessment of malignity-associated characteristics

Dedifferentiation is a hallmark of advanced cancer. A plethora of cellular markers allows assessing the differentiation state of hepatocytes and several studies have characterised the ten selected hepatoma cell lines [63, 64, 67, 68, 72]. Although nomenclature varies, there is an overall consensus discriminating the cell lines into better differentiated “epithelial-like” and poorly differentiated “fibroblast-like” (Table 1). This grouping fits well with the histological origin of the tumours: the tissue of HLE and HLF was described as “undifferentiated hepatocellular carcinoma” [70] and that of SNU182 and SNU387 as “poorly differentiated” HCC [71], with grades III and IV representing “embryonal-cell types” based on the Edmondson-Steiner’s classification [73].

Malignancies are defined as “diseases in which abnormal cells divide without control and can invade nearby tissues” [74]. In addition to the published information (Table 1), we therefore assessed three different parameters of the selected cell lines as a measure of functional malignity: cell migration, invasion and growth rate.

**Fig. 3:**
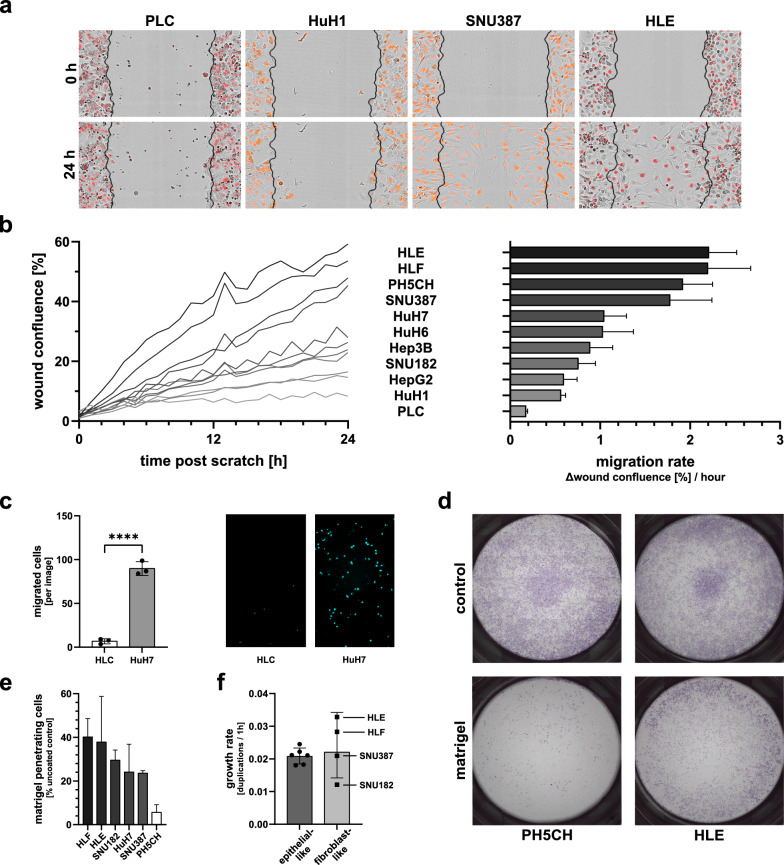
Functional assessment of the malignity of ten liver cancer cell lines. **a + b** Scratch wound migration assay. **a** Representative brightfield microscopy images; black lines: initial wound, red signal: nuclei. **b** Left: Cell confluence [%] in wound area over time. Right: Determination of migration rates (Δconfluence [%]/h) in the period 0–24 h by linear regression. **c** Migration of HLC and HuH7 through uncoated transwell membranes, Hoechst staining after 24 h. Left: Quantification. Right: Representative images (10 × magnification). **d + e** Transwell migration/invasion assay. **d** Representative membranes (matrigel coated, control: uncoated), stained with crystal violet 24 h after seeding. **e** Invasiveness, determined as the ratio of total cell count penetrating a matrigel-coated membrane vs. the corresponding uncoated membrane. **f** Proliferation rates (untreated). Panel (**b**) displays mean ± SEM, all other graphs mean ± SD of three independent repetitions

Regarding motility, we measured the cells’ ability to migrate into a scratch wound. Figure 3a shows exemplary images at fixed time points (0 and 24 h post scratching) for selected cell lines. PLC and HuH1 barely moved beyond the initial scratch margins, whereas the scratch was almost completely closed by HLE and SNU387 cells. We evaluated migration rates for all cell lines in live-cell imaging (Fig. 3b). Indeed, three of the four poorly differentiated cell lines, HLE, HLF and SNU387, were among the fastest moving cells. All epithelial-like cell lines exhibited a markedly reduced migration rate with PLC being the least motile cells. In general, we found a good correlation between reported differentiation status and migration. Only two cell lines were breaking this scheme–SNU182, described to be dedifferentiated, showed a migratory behaviour similar to the epithelial-like cell lines and PH5CH, believed to be non-malignant [45], exhibited high migration comparable to the dedifferentiated HLE and HLF cell lines (Fig. 3b). As PH5CH were immortalised by transduction with a viral oncogene, they might have acquired motility characteristics untypical for hepatocytes. As a more authentic and primary-like hepatocyte system, we therefore also tested hepatocyte-like cells (HLCs) that we differentiated from human derived induced pluripotent stem cells (iPSC) [47, 75]. In a transwell migration assay, we compared their migratory capacity to one of the epithelial-like hepatoma cell lines, HuH7. Indeed, terminally differentiated HLCs were hardly able to migrate through the porous matrix of the transwell, while HuH7, comparable to the scratch wound assay, showed robust motility (Fig. 3c). Remarkably, only one of the few iPSC-derived cells that migrated through the membrane, expressed the HLC differentiation marker albumin (Additional file 3: Fig. S3c), while the majority of cells that were able to migrate appeared to be fibroblast-like, a cell-type which is likely spontaneously co-differentiated along the HLC differentiation protocol [47, 75]. This confirmed that normal, non-malignant hepatocytes have no significant migratory potential, much akin to PLC.

While migration is essential for metastasis, another prerequisite of metastatic cells is their capacity to penetrate the basal membrane [76]. This feature, invasiveness, requires the expression and activation of extracellular proteases [77]. Hence, as a second measure of malignity, we assessed the cell lines’ invasiveness by quantifying their migration across a matrigel-coated transwell membrane in a chemotactic gradient. Reassuringly, despite their unexpected high motility, non-malignant PH5CH cells were only inefficiently able to cross the matrigel layer, showing the lowest quantifiable invasiveness in our assay (Fig. 3d, e). Furthermore, all four dedifferentiated (i.e. potentially highly malignant) cells lines, HLE, HLF, SNU387 and SNU182, were efficiently invading and transmigrating the membrane, underscoring their advanced cancerous stage (Fig. 3e). For SNU182, their comparably low migratory behaviour was reproduced in this assay, but their relative ability to traverse the matrigel layer indicates high invasiveness (compare Additional file 3: Fig. S3d, e). Furthermore, the high migration rate of HuH7 was confirmed and they exhibited a robust invasive capacity (see also Additional file 3: Fig. S3d, e). For the remaining epithelial-like cells, HuH6 and HuH1 did show invading cells, however migration was below our cut-off for quantification, and HepG2, Hep3B and PLC hardly had any successfully transmigrating cells.

Unrestricted cell growth is the defining feature of tumours, and clearly one of the prime selective advantages of individual cells during tumour evolution. We therefore determined proliferation rates of our cell lines as a third parameter in live-cell imaging. Interestingly, the well-differentiated epithelial-like cell lines exhibited moderate growth rates that were rather comparable to each other, with Hep3B marking the lower end (0.018 h^−1^) and HuH7 standing out at 0.025 h^−1^ (Fig. 3f). In contrast, proliferation rates differed vastly between the dedifferentiated fibroblast-like cell lines (Fig. 3f). Whereas SNU182 exhibited the slowest proliferation among all tested cell lines at 0.012 h^−1^, SNU387 grew comparably to the well-differentiated cluster by 0.021 h^−1^. Strikingly, HLE and HLF that already demonstrated the most pronounced migration and invasion, also proliferated at markedly higher rates by 0.033 h^−1^ and 0.028 h^−1^, respectively. Similar to migration, PH5CH also stood out with regard to proliferation, featuring the second-fastest proliferation rate among all tested cell lines at 0.091 h^−1^. Again, this is likely related to the expression of the large T-antigen.

In conclusion, despite malignity being a vaguely defined and multi-factorial feature, in our functional assays classical hallmarks of advanced (“aggressive”) tumour cells, such as cell migration, invasiveness and overall proliferation rate correlated well with the reported differentiation status of the cell lines (Table 2) [63]. Notable exceptions are HuH7, which proliferated and migrated at markedly higher rates than other epithelial-like cells, and SNU182, which–despite being classified as fibroblast-like–exhibited very slow proliferation and low motility. Furthermore, non-malignant, artificially immortalised PH5CH were among the most quickly proliferating cells and showed a high propensity to migrate. Hence, they cannot be regarded a proper reference for non-transformed hepatocytes in all respects.

**Table 2:** Summary of the cell lines’ functional malignant characteristics

Cell line	Migration rate [Δ wound confl. [%] / h]	Invasiveness	Proliferation [duplications per hour]	Relative growth in 8000 IU/ml IFN-β [%]
PH5CH	1.92 ± 0.58	6%	0.0324 ± 0.0016	53.28 ± 5.15
PLC	0.17 ± 0.04	^a^	0.0222 ± 0.0022	4.57 ± 9.13
HuH1	0.56 ± 0.10	^a^	0.0211 ± 0.0023	46.61 ± 9.54
HuH6	1.03 ± 0.59	^a^	0.0212 ± 0.0033	24.33 ± 21.77
HuH7	1.04 ± 0.44	> 20%	0.0245 ± 0.0048	23.93 ± 12.49
HepG2	0.59 ± 0.26	^a^	0.0183 ± 0.0044	55.31 ± 12.18
Hep3B	0.88 ± 0.44	^a^	0.0181 0.0017	69.67 ± 8.43
HLE	2.21 ± 0.54	> 35%	0.0328 ± 0.0026	89.38 ± 8.36
HLF	2.19 ± 0.83	> 35%	0.0283 ± 0.0046	86.24 ± 13.49
SNU182	0.75 ± 0.33	> 20%	0.0121 ± 0.0008	41.87 ± 21.08
SNU387	1.77 ± 0.81	> 20%	0.0210 ± 0.0007	42.62 ± 2.05

^a^count in uncoated control below cut-off (1000/membrane)

### Comparison of IFN-sensitivity across cancer cell lines

**Fig. 4:**
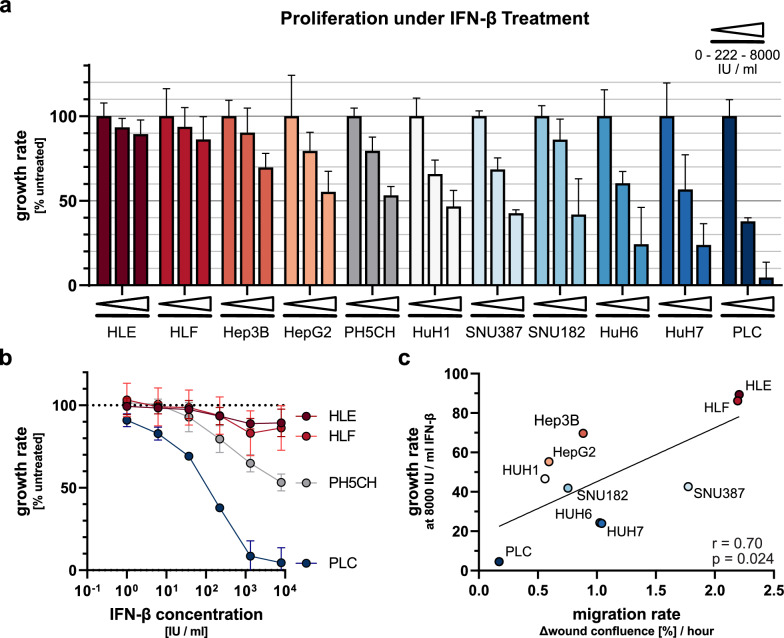
Antiproliferative effects of IFN-β on hepatoma cells. **a** Hepatoma cells were mock treated or stimulated with increasing doses of IFN-β (1–8000 IU/ml) and cell numbers were monitored for 96 h (imaged every 6 h) by live-cell imaging. Shown are growth rates normalised to untreated controls, for a medium (222 IU/ml) and the highest (8000 IU/ml) dose of IFN-β; see Additional file 4: Fig. S4 for all doses. **b** Full titration of HLE and HLF (dedifferentiated, high malignity), PLC (well differentiated, low malignity) and the control cell line PH5CH. **c** Relative growth rates at 8000 IU/ml IFN-β plotted against respective migration rates as determined before (Fig. 3b); plot against invasiveness see Additional file 3: Fig. S3f. Linear regression was performed and Pearson correlation was calculated. All graphs display mean (± SD in a and b) of three independent repetitions

According to our hypothesis, advanced tumours that developed in the context of an IFN-rich environment may be more resistant to growth inhibition by IFN. We, therefore, assessed the antiproliferative impact of increasing doses (0–8000 IU/ml) of IFN-β onto our panel of liver cancer cell lines. Indeed, IFN-β inhibited proliferation for all cell lines, however, to a substantially varying extent (Fig. 4a, Additional file 4: Fig. S4). While PH5CH showed the previously observed inhibition of roughly 50% at high doses, three of the well-differentiated hepatoma cell lines were affected even markedly stronger: HuH6, HuH7 and PLC, the latter of which exhibited an almost complete growth arrest at 8000 IU/ml IFN-β (Fig. 4a). On the contrary, the two fibroblast-like cells lines scoring highest in our malignity assays above (Table 2), HLE and HLF, proved profoundly resistant to IFN-β, with proliferation rates reduced by a mere 10–15% even at the highest dose (Fig. 4a). The different response profiles of PLC on one side and HLE and HLF on the other side of the non-malignant PH5CH is drastic (Fig. 4b). The effect of IFN-β on the remaining cell lines was more intermediate, with Hep3B showing least of an impact despite being a well-differentiated hepatoma cell line, and both SNU387 and SNU182 exhibiting a slightly stronger growth inhibition than PH5CH despite being fibroblast-like and, at least SNU387, higher malignity in our assays (Fig. 4a). Nonetheless, when plotting proliferation rates at 8000 IU/ml IFN-β over the respective capacity to migrate or invade, there appears to be an imperfect but statistically significant trend of more pronounced IFN-resistance in cells of higher malignity (p = 0.024, Pearson r = 0.70 for migration, Fig. 4c; p = 0.017, Pearson r = 0.94 for invasion, Additional file 3: Fig. S3f). This supports our hypothesis that IFN-resistance might be a feature more frequently found in advanced tumours that have undergone longer and/or stronger selection. Importantly, we do not propose that reduced sensitivity of cells towards IFN is causative or mechanistically linked to an increase in cell motility or invasiveness, but both might be consequences of more progressed tumour evolution. This is, for example, also supported by our observation that long-term selected PH5CH^IFN−β^ did exhibit reduced IFN-sensitivity (Fig. 2b, c), but did not exhibit an increased migratory phenotype (Additional file 2: Fig. S2f, g).

In summary, we found largely differing degrees of sensitivity to the antiproliferative effects of IFN-β across the studied panel of liver cancer cells. The fact that all investigated cell lines are of hepatocytic origin indicated that the observed differences cannot be due to cell type-specificity. Rather, they likely reflect the large heterogeneity typical for tumour cells, from which favourable phenotypes are selected and becoming dominant during tumour evolution. In fact, we found indications that particularly advanced tumours may develop resistance against growth inhibiting effects of IFN.

### Differences in IFN-signalling in hepatoma cells of high *vs.* low malignity

**Fig. 5:**
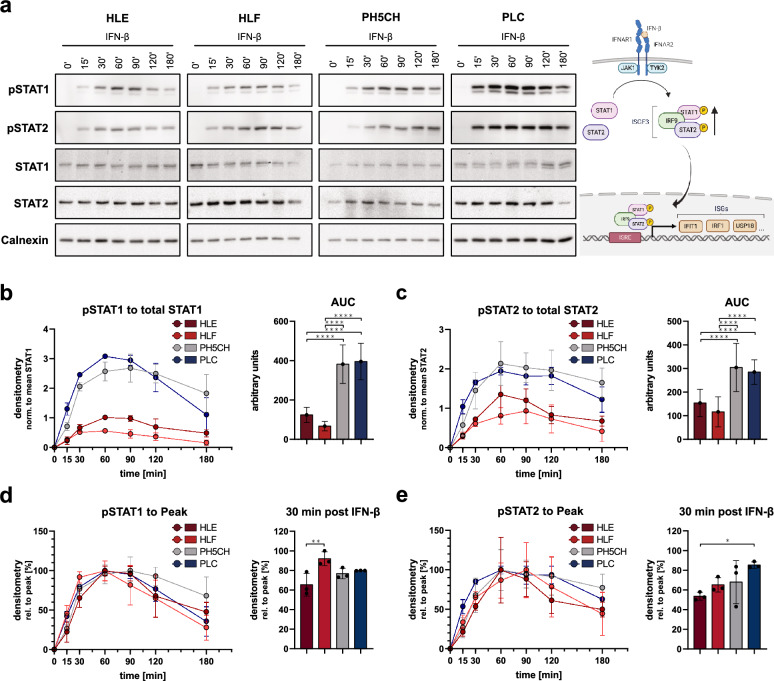
STAT1/2 phosphorylation upon stimulation with 130 IU/ml IFN-β. **a** Representative immunoblots of HLE, HLF, PLC and the non-malignant PH5CH control. Samples were adjusted for total protein content; calnexin levels vary between cell lines. Illustration on the right shows an overview of the IFN signalling pathway indicating all molecules assessed in the figure. **b** + **c** Quantified levels of phosphorylated STAT (pSTAT) relative to corresponding mean total STAT. Area under curve (AUC) used to quantify response over full time course. **d** + **e** Same data as in **b** + **c**, pSTAT normalised to its peak values (100%) to allow for better comparison of kinetics. All graphs display mean ± SD of three independent repetitions. Statistical analysis was performed with one-way ANOVA followed by Tukey’s multiple comparison test

The differences in the antiproliferative effect of IFN-β exposure across the tested cell lines could reflect general differences in the cell lines’ antiviral response and IFN system. Interestingly, however, we observed no major differences in replication of vesicular stomatitis virus (VSV) and the induction of an antiviral response (expression of the ISG IFIT1), with no correlation to the observed proliferative phenotypes (Additional file 5: Fig. S5a, b). Nonetheless, as antiproliferative phenotypes were strikingly different, we moved on to more closely investigate the cells’ IFN signalling pathway (scheme, see Fig. 5a), particularly with regard to its dynamics. The first major step in signal transduction downstream of the IFN-receptor (IFNAR) complex is phosphorylation of STAT1 and STAT2. For the further experiments, we focussed on the most lowly (HLE, HLF) and highly sensitive cells (PLC), as well as the non-malignant control PH5CH exhibiting an intermediate phenotype (Fig. 4). We stimulated them with a moderate dose of IFN-β (130 IU/ml) and assessed STAT1 and STAT2 phosphorylation in a time course up to three hours (Fig. 5). We normalised the phospho-specific signal to the total level of the respective STAT; of note, the overall expression of both STATs, being ISGs themselves, did not change significantly over the course of the experiment. Interestingly, while the dedifferentiated cell lines HLE and HLF were generally responsive to IFN treatment (see also Additional file 5: Fig. S5a, b), phosphorylation of STAT1 and STAT2 was significantly higher in PLC and the PH5CH control (Fig. 5b, c). This striking difference in the degree of phosphorylation was not reflected in the kinetics of phosphorylation. In both HLE/HLF and PLC (as well as PH5CH) STAT phosphorylation occurred already at 15 min and reached peak levels at 60–90 min (Fig. 5d, e). For STAT2, phosphorylation in PLC appeared to be slightly faster than in the other cell lines (Fig. 5e). Taken together, despite general functionality of the IFN signalling cascade in both HLE/HLF and PLC, we observed a substantially lower degree of STAT phosphorylation upon IFN treatment of HLE and HLF cells, mirroring their significantly reduced antiproliferative response.

We next investigated the impact of reduced STAT phosphorylation on the induction kinetics of ISGs. Therefore, we measured the expression of three ISGs upon IFN-β treatment in a time course over 24 h: (1) IFIT1, one of the strongest induced type I ISGs; (2) USP18, a major negative feedback regulator of type I IFN signalling that has previously been implicated with antiproliferative effects; and (3) IRF1, a major ISG of the type II IFN response mediating growth inhibiting effects of IFNγ [78]. IRF1 is also induced downstream of the type I IFN receptor (IFNAR) by STAT1-STAT1 homodimers [79]. Importantly, it is a so called tuneable ISG, meaning that its induction is less switch-like but increases gradually with duration and intensity of the IFN signal [80], reminiscent of what has been described for the antiproliferative effects [24]. We furthermore included HuH1 in the experiments as a representative of well-differentiated malign (as opposed to non-malign PH5CH) cell lines with intermediate growth inhibition by IFN-β (Fig. 4a).

**Fig. 6:**
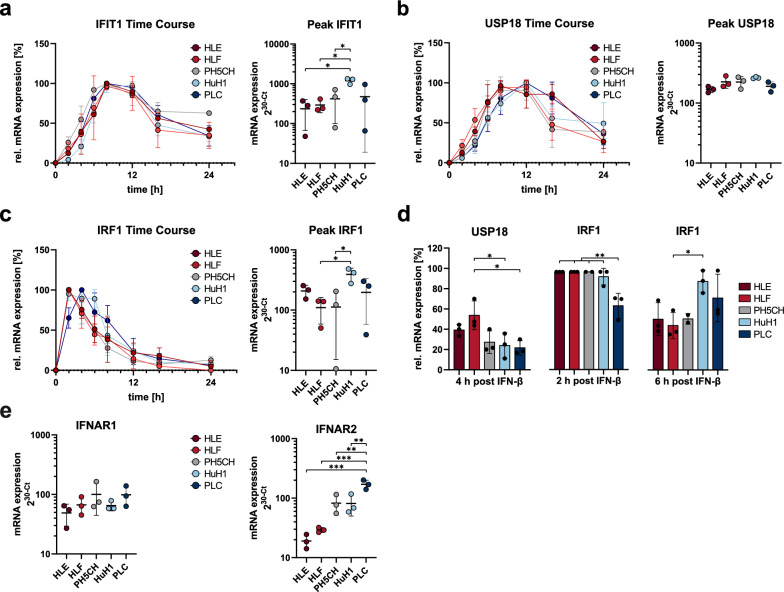
Induction of ISG mRNA upon stimulation with 130 IU/ml IFN-β. **a–c**: Left panels: kinetics displayed as expression levels normalised to peak values. Gross outliers were excluded (IFIT1: HLE, PH5CH, PLC; IRF1: PH5CH; n = 2). Right panels: absolute expression levels determined at peak time points. **d** Relative expression levels at selected time points. **e** Type I IFN receptor mRNA expression in untreated cells. All graphs display mean ± SD of three independent repetitions (exceptions noted above). Absolute expression levels were determined independently of housekeeping genes as 2^30−Ct^/4.2 ng of total RNA. Statistical analysis was performed with one-way ANOVA followed by Tukey’s multiple comparison test

As already observed upon VSV infection (Additional file 5: Fig. S5b), all five tested cell lines showed comparable induction of IFIT1 and, strikingly, expression kinetics were virtually identical (Fig. 6a). Peak levels were only slightly lower in HLE and HLF (reaching significance only versus HuH1; Fig. 6a, right panel). In case of the negative regulator USP18, induction occurred earlier in HLE and HLF (Fig. 6b), resulting in slightly but reproducibly higher mRNA expression at 4 h (Fig. 6d). Peak induction, however, was comparable between all five cell lines (Fig. 6b, right panel). This faster USP18 induction might contribute to a quicker and stronger dampening of IFN responses in HLE and HLF, however it has been reported to hardly affect IFN-β signalling [81]. Lastly, for IRF1 we found a very steep and early induction with a peak already at 2 h post treatment for HLE and HLF, but also HuH1 and PH5CH, whereas PLC reached a peak only by 4 h (Fig. 6c, d). IRF1 expression was very transient and exhibited a rather rapid decay in HLE, HLF and PH5CH, while it was somewhat more sustained in HuH1 and particularly in PLC. Together with higher peak expression levels (Fig. 6c, right panel), this led to appreciably higher IRF1 expression at 6 h in HuH1 and PLC (Fig. 6d). The higher and particularly longer lasting expression of this transcription factor might contribute to the strong antiproliferative impact of IFN on PLC. Nonetheless, we were overall surprised to see rather minimal differences in the induction kinetics of these three different classes of ISGs. This suggested the strong difference in the cell lines’ proliferative response to IFN-β might in fact not be due to canonical antiviral transcriptional responses.

As clear differences in terms of STAT1/2 activation were evident, we hypothesised the differential response of the cell lines might originate already at the receptor level. Hence, we analysed the mRNA expression levels of the two receptor subunits, IFNAR1 and IFNAR2 (Fig. 6e). For IFNAR1, we found a slight trend towards lower expression in HLE and HLF. For IFNAR2, this trend was substantially stronger, with PLC expressing approximately tenfold higher levels of IFNAR2 as compared to HLE. In analogy, expression was markedly higher in PH5CH and HuH1, both being much more susceptible to growth inhibition by IFN-β than HLE or HLF.

We could not clearly identify the underlying mechanism for the profoundly different antiproliferative response of the hepatoma cell lines upon IFN-β treatment. The most pronounced difference was observed for the mRNA expression levels of the IFN receptor chains, in particular IFNAR2. This translated into significant differences in STAT1/2 phosphorylation between HLE/HLF and the less malign PH5CH and PLC. Curiously, these differences were apparently overridden downstream in the signalling cascade, as induction kinetics and strength for three very different ISGs–IFIT1, USP18 and IRF1–were remarkably similar.

### Differentially expressed factors in IFN-β-resistant hepatocytes

**Fig. 7:**
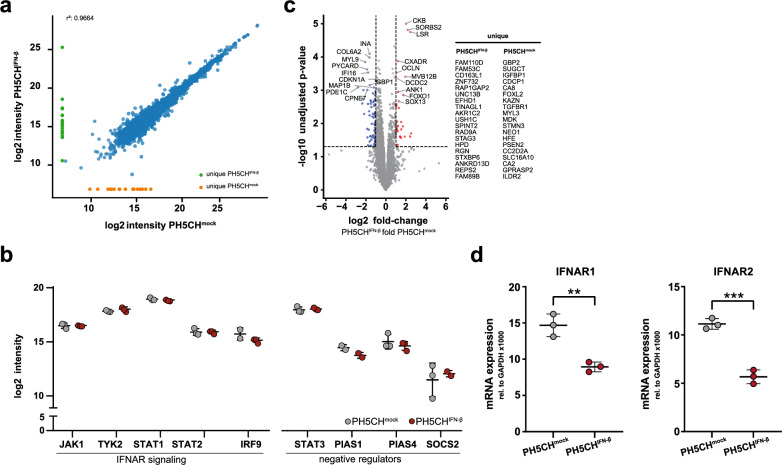
Comparison of gene expression in PH5CH^mock^ and PH5CH^IFN−β^ cells (compare Fig. 2). **a–c** Protein expression levels of PH5CH^mock^ and PH5CH^IFN−β^ cells determined by label-free mass-spectrometry-based proteomics. Cells were analysed nine days after withdrawal of IFN-β. **a** Scatter plot comparing log_2_-transformed protein intensity for each protein in in PH5CH^IFN−β^ compared to PH5CH^mock^ cells. Green and orange dots represent proteins only detected in one cell population. **b** Dot-plot depicting the log_2_-transformed protein intensity of selected key members and regulators of type I IFN signalling. Grey dots represent PH5CH^mock^ and red dots represent PH5CH^IFN−β^ **c** Volcano plot illustrating significantly differentially expressed proteins in PH5CH^mock^ and PH5CH^IFN−β^ cells. The -log_10_-transformed unadjusted p-value is plotted against the log_2_ PH5CH^IFN−β^/PH5CH^mock^ fold-change. The dotted horizontal line denotes unadjusted p-value < 0.05 and the dotted vertical lines a fold change cut-off of twofold. 6974 proteins were detected in both samples, blue dots indicate proteins that are more abundant in PH5CH^mock^ cells (81 proteins), red dots indicate proteins that are more abundant in PH5CH^IFN−β^ cells (29 proteins). Proteins uniquely identified in either condition given in the list on the right. **d** mRNA expression levels of IFNAR1 and IFNAR2, determined by qRT-PCR. Cells were analysed 29 days after withdrawal of IFN-β. All graphs display mean (± SD) of three subpopulations of one PH5CH^IFN−β^ population and PH5CH^mock^

We could roughly classify the hepatoma cell lines used in this study according to their degree of malignity (Fig. 3). Nonetheless, they stem from completely different, i.e., genetically diverse individuals (exception: HLE and HLF), and their tumour evolution occurred under unknown physiological constraints and selection pressures. Hence, identifying concrete and common molecular mechanisms explaining resilience towards the antiproliferative action of IFN-β may be very challenging. We therefore made use of our in vitro selection assay, in which we subjected non-neoplastic PH5CH to continuous IFN-β stimulation (Fig. 2). As in this setting the genetic background of IFN-sensitive PH5CH^mock^ and IFN-β-resistant PH5CH^IFN−β^ is identical and we applied one single strong and defined selection pressure, expression differences may have a substantially higher likelihood to be causal for the observed phenotype. We therefore subjected these two populations of PH5CH to quantitative full proteome mass spectrometry. As expected, the two populations were overall highly similar (Fig. 7a). We specifically analysed central components of the IFN signalling pathway. Unfortunately, not all proteins were detected by this unbiased full proteome approach. Still, among the successfully detected proteins were key components such as the kinases JAK1 and TYK2 as well as transcription factors STAT1, STAT2 and IRF9 (not detected in all replicates). For none of them we found statistically significant differences in protein levels (Fig. 7b). Furthermore, putative negative regulators of IFN signalling, such as STAT3 [82], SOCS2, PIAS1 and PIAS4, were expressed to virtually identical levels (Fig. 7b). Nonetheless, global analysis of the proteomes did reveal statistically significant differences in expression of several proteins (Fig. 7c); none of them, however, is a canonical member of IFN signalling. We performed global pathway analyses on the differentially expressed proteins (Additional file 6: Fig. S6, Additional file 7), but future follow-up studies will be required to scrutinise the potential involvement of identified pathways in IFN signalling.

Lastly, we wanted to assess the expression levels of the type I IFN receptor components, as it was the most striking finding in HLE and HLF cells (Fig. 6e). Unfortunately, detecting protein levels of IFNAR1 and IFNAR2 is very challenging, both by antibody-based methods as well as mass spectrometry. We, therefore, assessed mRNA levels by qRT-PCR. Indeed, there was a clear difference in mRNA expression, with both IFNAR1 and IFNAR2 being significantly downregulated in the IFN-resilient PH5CH^IFN−β^ population–even at four weeks after withdrawal of IFN-β selection (Fig. 7d). As this remarkably resembled the expression pattern in HLE/HLF and PLC, we lastly assessed IFNAR levels across all ten liver tumour cell lines. Both, IFNAR1 and IFNAR2 expression varied roughly five- to tenfold across the cell lines, but only IFNAR2 levels correlated highly significantly with the antiproliferative effect of IFN-β treatment (Additional file 5: Fig. S5e, f).

In summary, we performed full proteomic comparison of non-selected PH5CH^mock^ and PH5CH^IFN−β^ that underwent 6 weeks of selection under continuous presence of IFN-β. None of the differentially expressed proteins was directly involved in canonical IFN signalling, and all IFN pathway components we checked explicitly were comparably expressed between the two conditions. At the mRNA level we found IFNAR1 and IFNAR2 to be significantly less expressed in PH5CH^IFN−β^, recapitulating our findings in HLE and HLF. Moreover, across all ten examined hepatic tumour cell lines we observed a highly significant correlation of IFNAR2 expression with sensitivity to the antiproliferative effect of IFN-β. This suggests IFNAR expression may vary substantially across individual cells and these expression differences are stable over time, putatively through epigenetic mechanisms. Therefore, cells with low receptor expression can be selected for under prolonged presence of IFN-β and maintain their phenotype at least for several weeks after removal of the selective pressure.

## Discussion

Type I IFNs are cytokines first produced and secreted by virus infected cells as part of the cell-intrinsic antiviral immunity, and later on by innate immune cells, such as dendritic cells. They signal autocrine and–arguably more importantly–paracrine, eliciting a very strong transcriptional response in recipient cells. Beside their critical role in impeding virus replication, IFNs have also long been described to exhibit cytostatic or even cytotoxic effects, particularly in tumour cells but also normal tissue [3–5]. In fact, IFNs have been proposed as strong anticancer agents for more than 60 years [3, 83], although the underlying mechanisms are complex and not comprehensively understood. Type I IFN clearly impacts tumour cells themselves in various ways, also beyond direct cytostatic effects, for example by suppressing metastases through the upregulation of E-cadherin [84–87]. IFNs furthermore exhibit strong immunomodulatory functions shaping the tumour microenvironment (TME). However, depending on the context, even tumour promoting functions have been described [28]. This highlights the urgent need to better understand the molecular effects of IFNs, in order to optimally exploit them for clinical use, e.g. in cancer immune therapy [29, 83].

We hypothesise that the antiviral system is an essential part of the early recognition of carcinogenic events and IFN plays a key role in the control of malignantly transformed cells and tumour growth. In fact, we and others have previously described the involvement of antiviral pathways in the detection of genotoxic insults (DNA damage) and the appropriate induction of cell death [31–33, 88]. It has further been well documented in various experimental systems that IFN-deficiency can promote tumour development and growth [28, 29]. While professional cellular immunity has clearly been implicated in this phenomenon, IFN production and signalling furthermore have a decisive direct impact on cell viability and proliferation. Hence, we speculate that this poses a strong selective pressure on actively dividing tumour cells. In a somewhat broader sense, also such innate immune responses can be considered immune pressure and the expansion of tumour subclones exhibiting increased resistance towards adverse, antiproliferative effects of IFN can be viewed as immunoediting. This has indeed been described previously, and in certain instances even IFN-adaptation and -dependence was observed, in which tumours “learned” to exploit protumourigenic features of ISGs while overcoming antiproliferative effects [30, 44, 89–91].

In our present study, we addressed this hypothesis of a strong selective pressure on proliferating cells in a type I IFN-rich milieu as one driving force of immunoediting. We approached this from two angles: first, we reproduced the selective pressure by IFN-β in vitro and assessed whether IFN-resistance emerges over time; and second, we tested ten different tumour cell lines from hepatocellular carcinomas of different stages with regard to their insensitivity towards the antiproliferative effects of IFN-β. In the latter approach, we hypothesize that IFN-resistance is a selected trait and, hence, would be more pronounced in more progressed tumour stages. As for the in vitro approach, we first confirmed the postulated proliferative advantage of IFN-resistant cells in a mixed but genetically homogeneous population of sensitive (wildtype) and resistant (IFN receptors KO) cells. Moreover, by exposing non-malignant PH5CH hepatocytes to high concentrations of IFN-β continuously over the course of several weeks, we observed the emergence of a less sensitive phenotype (PH5CH^IFN−β^) with respect to the antiproliferative effects of IFN. This demonstrates the feasibility of the hypothesis of immunoediting due to selective proliferative pressure by these cytokines. As for the underlying mechanism, it is interesting to note that the resistant phenotype reverted only very slowly and could still be observed even four weeks after IFN-withdrawal. This argues either for the selection of genetic variants, which appears unlikely given the non-neoplastic origin of the cell line, or epigenetic effects. Comparing these PH5CH^IFN−β^ to a non-selected control using full-proteome mass spectrometry, we could not find significant differences in the detected canonical IFN pathway members, and an overall highly similar proteome. Nonetheless, it will be interesting to look into the differentially expressed proteins and their role in IFN-mediated growth inhibition in follow-up studies. For example, one of the most important regulators of cell cycle progression, CDKN1A (*aka* p21), was found to be significantly downregulated in PH5CH^IFN−β^, as were IFI16 and GBP1, both of which have been implicated in modulating cell proliferation [92, 93]. We furthermore performed global pathway analyses, but no single process stood out that could immediately explain the observed IFN-insensitivity; further research will also be needed in this regard.

For our second line of experiments, we analysed IFN-sensitivity of ten different liver tumour cell lines. Liver cancer frequently develops on a background of hepatitis, a chronic inflammatory condition, and as such might have higher exposure to inflammatory cytokines and IFNs. Of note, it has been shown that diffusion ranges of cytokines (and interferons in particular) in a 3D tissue context are extremely limited (to ~ 100 µm), suggesting high locally acting concentrations [94, 95]. Particularly for chronic HCV infection, continuous production of type I IFN in the liver has been established with strong induction of hepatic ISG expression [39–41], which again suggests high local concentrations, making those tumours putatively the most likely to have undergone strong IFN-driven selection. Most cell lines were established in Japan before the identification of HCV, but notably, later epidemiologic studies from Japan point towards a high prevalence at the time of cell isolation, rendering an HCV-association of the HBV-negative tumours of HLE, HLF and HuH7 very conceivable. Several other cell lines derive from tumours that clearly emerged in the context of HBV infection, with detectable HBV DNA-integrates and, in case of PLC and HuH1, ongoing secretion of the HBs antigen (see Table 1). The ten cell lines can be classified as well-differentiated (epithelial-like morphology), and putatively further advanced, dedifferentiated tumours (fibroblast-like morphology) [63]. We could, at least in part, confirm this functionally by analysing typical malignity-associated features such as proliferation rate, propensity to migrate (wound healing) and invasion capacity (i.e., ability to penetrate matrigel-coated membranes). Upon treatment of these cell lines with increasing concentrations of IFN-β, we found striking differences in terms of its antiproliferative effect. In particular HLE and HLF, standing out with their functional features of malignity, exhibited an almost complete resistance to growth inhibition by IFN-β (max reduction of growth rate by 10–15%). In contrast, the well-differentiated PLC, for which we found little to no migratory and invasive capacity, were extremely sensitive to IFN treatment (max reduction of growth rate by 95%). Overall, we found a significant correlation between the invasion capacities as well as migration rates of cell lines and their IFN-β sensitivity. However, for migration, this correlation was largely driven by the extremes HLE and HLF on the one end, and PLC on the other end. Of note, HLE and HLF were derived from the same patient’s tumour [70], further reducing the interpretability of this correlation. Moreover, a previous study analysing growth inhibitory effects of type I IFN across different HCC-derived cell lines did not observe a general correlation with the histological grade of the original tumours [96]. Nonetheless, due to intratumoural heterogeneity, this question is hard to tackle using clonal cell lines. Another notable aspect is the phenotype of PH5CH used as a non-malignant control. PH5CH are derived from non-neoplastic peritumour liver tissue of an HCV-positive HCC patient, and were shown to have a wildtype p53 locus (in contrast to tumour-derived cells from the same patient) [45]. They are used in various studies as non-transformed hepatocyte controls [97, 98], however it must be noted that they were immortalised by stable transfection of the SV40 large T-antigen (LTAg) [45]. LTAg is known to interact with and inactivate the tumour suppressors Rb and p53, which has also been implicated in tumour cell motility and invasion [99–101]. In line with this function of LTAg, we found PH5CH to exhibit a high proliferation and migration rate as well as a low but detectable capacity to invade in a matrigel transwell assay, which one would not expect from physiological hepatocytes. Indeed, iPSC-derived hepatocyte-like cells (HLC) [47] did not show notable motility in our hands. Nonetheless, PH5CH were still our best experimentally amenable control for well-differentiated hepatocytes that have not undergone malign transformation. In terms of the antiproliferative effect of IFN-β, they also proved to be substantially more sensitive than HLE and HLF (max reduction of growth rate by 50%), comparable to the majority of the well-differentiated hepatoblastoma and HCC cell lines.

We performed further analyses in order to understand the mechanism behind the drastic differences between the virtually IFN-resistant HLE and HLF, the IFN-sensitive control PH5CH, and particularly the highly sensitive PLC cells. We observed astonishingly little differences in the induction of the classical ISGs IFIT1, USP18 and IRF1. While there was a reproducible trend of lower peak levels in HLE and HLF, particularly so for IFIT1 and IRF1, this indicated that the canonical antiviral downstream signalling of IFN was overall intact. This was furthermore confirmed in VSV infection, which in all ten cell lines triggered a similar ISG response. The most striking difference in terms of IFN signalling was observed for the phosphorylation of STAT1 and STAT2, for which we found a very clear segregation between HLE/HLF on the one hand, and PH5CH and PLC on the other. This argues for an effect at the level of the receptor complex comprising also the kinases phosphorylating the STATs. In fact, we found substantially lower expression levels of IFNAR2 in HLE and HLF as compared to PH5CH and HuH1, but particularly to PLC, perfectly mirroring those cell lines’ antiproliferative response to IFN-β treatment. This further held true when assessing IFNAR expression in all ten cell lines, which specifically for IFNAR2 correlated highly significantly with their sensitivity towards the antiproliferative effect of IFN-β. This supports previous studies establishing an association of IFN receptor levels with the antiproliferative efficacy of IFN in HCC [27, 102], and is in line with reports on IFNAR downregulation in advancing bladder cancers [103], and also BRAF-mutated melanoma [104]. Corroborating the IFNARs as sensitive cellular targets for modulating sensitivity to cell-growth inhibition by IFN-β, also the in vitro selected PH5CH^IFN−β^ exhibited a striking downregulation of IFNAR1 and particularly IFNAR2. Also in another human liver cancer cell line, downregulation of IFNAR2 was previously reported upon long-term exposure to type I IFN, however with no clear correlation to proliferation or cell viability [96]. Altogether, these are strong indications that expression specifically from the IFNAR2 locus may be particularly amenable to modulation, possibly at an epigenetic level. Indeed, it has been reported that the IFNAR promoters can be regulated by methylation [105], suggesting DNA hypermethylation could be a possible mechanism of IFNAR downregulation. While this will require further studies, our proteomics approach found PYCARD, a sensitive marker for promoter hypomethylation by DMNT1 [106], to be one of the (few) significantly downregulated proteins in PH5CH^IFN−β^.

As for the molecular mechanism of how IFNAR2 levels modulate specifically the antiproliferative effect of IFN-β, it is interesting to note that these differences at the receptor level did not translate into clearer differences in the transcriptional induction of ISGs. In general, ISGs can be classified into tuneable *versus* robust genes, whereby the expression of tuneable genes is highly dependent on receptor density, duration of stimulation and receptor binding affinity, whereas robust ISGs are fully induced already upon minute stimulation [24, 107]. Interestingly, antiproliferative effects were reported to be predominantly mediated by tuneable genes [108–110], whereas some of the robust ISGs, such as the IFIT family, including the prototypical IFIT1, have recently been implicated even with pro-oncogenic effects [111]. We assessed IRF1 as a reportedly tuneable ISG [80] with antiproliferative and tumour-suppressor function [112, 113]. Surprisingly, also for IRF1 the difference in the level of transcriptional induction was very modest, however, it appeared to show slightly more sustained expression in PLC cells. Another candidate for mediating antiproliferative or even pro-apoptotic effects of IFN-β is the USP18/ISG15 system. USP18 is a well-known negative feedback regulator of IFNAR signalling, but in non-induced, homeostatic conditions was reported to positively regulate cell cycle progression and even promote tumour growth and progression across different models [114–116]. Interestingly, in the IFN system it functions by being recruited to IFNAR2 and competing with the kinase JAK1 for binding to the IFNAR complex [117]. This inhibitory function in IFN signalling is independent of its homeostatic function and its enzymatic activity as an ISG15-specific isopeptidase [118]. Hence, it is tempting to speculate that reduced expression of IFNAR2 as we observed in the highly malignant cell lines still permits the efficient transcriptional induction of ISGs, including USP18 (as we could show), while the lower IFNAR2 levels might lead to less sequestration of USP18 to the IFN receptor complex and, consequently, increased cytosolic availability and proproliferative activity. The USP18/ISG15 system also appears to be the main determinant for the differential effects of IFN-α (lowly antiproliferative) versus IFN-β (strongly antiproliferative) [119], further supporting this hypothesis. Future mechanistic investigation, however, will be required to corroborate this model experimentally.

Our study demonstrated the impact of type I IFN on the proliferative status of cells, particularly in the context of cancer cells. It clearly supports the hypothesis of IFN-driven immunoediting at the cancer cell-intrinsic level, and establishes IFNAR2 as a sensitive target. A major limitation, however, is that we could not investigate tumour cell-extrinsic effects of IFN, such as those on angiogenesis and immune cell regulation, which clearly play an important role, too [6–14]. It becomes increasingly clear that IFNs exert a multitude of effects in the organism, some of which are directly (e.g. the here investigated tumour cell-intrinsic antiproliferative effect) or indirectly (e.g. anti-angiogenetic effects) impacting tumour development, whereas others may even promote tumour growth and cancer progression. This may be dependent on the organ and cell type of origin of the tumour, as well as the, particularly immunological, composition of the TME and lastly also on the individual clonal evolution of the tumour. In our study, we have focused on the direct, tumour cell-intrinsic impact of IFN-β on cell proliferation only. While we do recognise certain, possibly generalisable, features, the complexity of effects requires further experimental scrutiny, both, to reach a better understanding of the impact of the endogenous IFN-system on cancer cell surveillance and control, as well as to direct the development of parameters and biomarkers allowing the stratification of putatively IFN-responsive versus non-responsive tumours for individualised oncological therapies.

### Supplementary Information

**Additional file 1: Figure S1:** Fluorescence microscopy images during selection experiment. 5% or 10% (as indicated) of IFN-β unresponsive IFNAR DKO A549 cells (nuclear H2B-mCherry, magenta) were cocultivated with control A549^NT^ cells (cytoplasmatic GFP, cyan). IFNAR1 KO gave a relative advantage to resistant cells in conditions continuously stimulated with 5000 IU/ml IFN-β over mock.

**Additional file 2: Figure S2:** Comparison of distinct populations of PH5CH that were selected by continuous IFN-β pressure.** a** Interim analysis of IFN-β-inhibited cell growth after 3, 4 or 6 weeks of selection and one week of recovery. Relative growth of PH5CH^IFN−β^ vs. PH5CH^mock^ upon stimulation with low (100–222 IU/ml) or high dose (1000–1333 IU/ml) of IFN-β. **b-e** Three separate populations of PH5CH (PH5CH^IFN−β 1–3^) were selected by continuous IFN-β pressure (2000 IU/ml) over six weeks (see Fig. 2). Growth rates determined after one and four weeks of recovery and compared to a population passaged in parallel in absence of IFN-β (PH5CH^mock^) and/or a freshly thawed population of PH5CH (PH5CH^naïve^). **b + d** Growth rates relative to mock at different IFN-β concentrations. Untreated condition is represented at x = 10^0^ IU/ml for simplicity. PH5CH^naïve^ were thawed 1 week before the experiment and behaved very comparable to PH5CH^mock^, whereas PH5CH^IFN−β^ populations were less impaired in growth by IFN-β. **c + e** Absolute growth rates in mock condition vs. stimulation with 8000 IU/ml IFN-β. **f + g** Migration rate of PH5CH^mock^ and PH5CH^IFN−β 1–3^ determined in scratch wound assay. **f** Representative microscopic images. Wound margins at 0 h are demarcated in black, nuclei in orange. **g** Migration rate calculated as in Fig. 3 during period of highest motility (0–12 h). Graphs display mean ± SD.

**Additional file 3: Figure S3:** Assessment of immune status and malignity of ten liver cancer cell lines.. **a** Levels of secreted HBs antigen in the supernatant of five hepatoma cell lines from our panel that have been reported to harbour integrates of HBV were measured using The ARCHITECT HBsAg assay. **b** Expression of IFN-β at the basal level shown as absolute mRNA levels. The graph displays mean (± SD) of three separate replicate wells, except for the Hep3B, SNU182 and SNU387 cell lines, as indicated. Note all cell lines showed signals around C_T_ = 30 being close to the detection limit. No IFN-β protein was detected in supernatants by ELISA. **c** HLC on uncoated membranes 24 h after seeding. No scrub (upper images) visualises all HLC cells. Hoechst and albumin staining show differentiation into HLC. After scrubbing (lower images), only cells that migrated to the lower chamber remain. The arrow points out a single albumin expressing cell on the lower surface, while other migrated cells were albumin negative (not hepatocyte-like). Representative images (20 × magnification). **d + e**: Total counts of cells that migrated through uncoated (**d**) respectively matrigel-coated (**d**) transwell membranes after 24 h. Fast migrating PH5CH, HLE, HLF and SNU387 were seeded at 2.5 × 10^4^, other cell lines at 1 × 10^5^ cells/chamber. **f** Relative growth rates at 8000 IU/ml IFN-β plotted against respective invasiveness of cell lines showing > 1000 migrated cells on uncoated membrane as determined in Fig. 3. Linear regression was performed and Pearson correlation was calculated.

**Additional file 4: Figure S4:** Growth inhibition of hepatoma cells by increasing concentrations of IFN-β. Mock stimulation is represented at x = 10^–1^ IU/ml for simplicity. IC_50_ included as orientation (dashed vertical line). Graphs display mean ± SD of three independently repeated experiments, each consisting of 12 technical replicates (4 images from 3 separate wells per condition). Determination of growth rates as described in methods.

**Additional file 5: Figure S5:** Hepatoma cell lines have an intact response to viral infection and the differential antiproliferative effect of IFN-β negatively correlates with the expression of baseline levels of IFNAR2. **a + b** Hepatoma cells (3.5 × 10^5^) were mock treated or infected with VSV-MQ recombinant virus expressing eGFP at an MOI of 10. After 24 h, cells were analysed via qRT-PCR for their permissiveness to viral replication (**a**) and mounting of an antiviral response (**b**). **c + d** Expression levels of IFNAR1 and IFNAR2 subunits quantified independently of the housekeeping genes. **e + f** Relative growth rates at 8000 IU/ml IFN-β plotted against IFNAR1 (**e**) or IFNAR2 (**f**) expression. Pearson correlation and linear regression was calculated. All graphs display mean (± SD) of three separate replicate wells, except for Hep3B, SNU182 and SNU387 cell lines, as indicated.

**Additional file 6: Figure S6:** Long-term selected PH5CH cells were subjected to full-proteome mass spectrometry analysis and differentially expressed proteins (PH5CH^IFN−β^ versus PH5CH^mock^) were analysed by IPA pathway analysis (Qiagen). For full IPA analysis report, see Additional file 7.

**Additional file 7.** IPA analysis report.pdf. This file contains the full report of the Qiagen IPA analysis of the proteomes shown in Additional file 6: Fig. S6

## Data Availability

The mass spectrometric raw data as Spectronaut files (PH5CH^mock^ and PH5CH^IFN−β^) are available on the proteomeXchange PRIDE platform [52] http://www.proteomexchange.org, under the accession number PXD043761. All further raw data will be available upon request from the corresponding author: m.binder@dkfz-heidelberg.de.

## References

[1] Isaacs A, Lindenmann J (1957). *Virus interference*. I. The interferon. Proc R Soc Lond B Biol Sci.

[2] Schneider WM, Chevillotte MD, Rice CM (2014). Interferon-stimulated genes: a complex web of host defenses. Annu Rev Immunol.

[3] Paucker K, Cantell K, Henle W (1962). Quantitative studies on viral interference in suspended L cells. Virology.

[4] Taylor-Papadimitriou J (1985). Antiviral and antiproliferative effects of interferons in quiescent fibroblasts are dissociable. Virology.

[5] Clemens MJ, McNurlan MA (1985). Regulation of cell proliferation and differentiation by interferons. Biochem J.

[6] Enomoto H (2017). The in vivo antitumor effects of type I-interferon against hepatocellular carcinoma: the suppression of tumor cell growth and angiogenesis. Sci Rep.

[7] Sidky YA, Borden EC (1987). Inhibition of angiogenesis by interferons: effects on tumor- and lymphocyte-induced vascular responses. Cancer Res.

[8] Rosewicz S (2004). Interferon-alpha: regulatory effects on cell cycle and angiogenesis. Neuroendocrinology.

[9] Ma Z, Qin H, Benveniste EN (2001). Transcriptional suppression of matrix metalloproteinase-9 gene expression by IFN-gamma and IFN-beta: critical role of STAT-1alpha. J Immunol.

[10] Zheng H (2011). Vascular endothelial growth factor-induced elimination of the type 1 interferon receptor is required for efficient angiogenesis. Blood.

[11] Medrano RFV (2017). Immunomodulatory and antitumor effects of type I interferons and their application in cancer therapy. Oncotarget.

[12] Yang I (2004). Modulation of major histocompatibility complex Class I molecules and major histocompatibility complex-bound immunogenic peptides induced by interferon-alpha and interferon-gamma treatment of human glioblastoma multiforme. J Neurosurg.

[13] Gessani S (2014). Type I interferons as regulators of human antigen presenting cell functions. Toxins.

[14] Fenton SE, Saleiro D, Platanias LC (2021). Type I and II interferons in the anti-tumor immune response. Cancers.

[15] Murphy D (2001). Interferon-alpha delays S-phase progression in human hepatocellular carcinoma cells via inhibition of specific cyclin-dependent kinases. Hepatology.

[16] Murata M (2006). A comparison of the antitumor effects of interferon-alpha and beta on human hepatocellular carcinoma cell lines. Cytokine.

[17] Sangfelt O (1999). Molecular mechanisms underlying interferon-alpha-induced G0/G1 arrest: CKI-mediated regulation of G1 Cdk-complexes and activation of pocket proteins. Oncogene.

[18] Yano H (1999). Interferon alfa receptor expression and growth inhibition by interferon alfa in human liver cancer cell lines. Hepatology.

[19] Chen J (2016). The cell-cycle arrest and apoptotic functions of p53 in tumor initiation and progression. Cold Spring Harb Perspect Med.

[20] Sangfelt O, Strander H (2001). Apoptosis and cell growth inhibition as antitumor effector functions of interferons. Med Oncol.

[21] Mandal M (1998). Interferon-induces expression of cyclin-dependent kinase-inhibitors p21WAF1 and p27Kip1 that prevent activation of cyclin-dependent kinase by CDK-activating kinase (CAK). Oncogene.

[22] Takaoka A (2003). Integration of interferon-alpha/beta signalling to p53 responses in tumour suppression and antiviral defence. Nature.

[23] Carvajal Ibanez D (2023). Interferon regulates neural stem cell function at all ages by orchestrating mTOR and cell cycle. EMBO Mol Med.

[24] Piehler J (2012). Structural and dynamic determinants of type I interferon receptor assembly and their functional interpretation. Immunol Rev.

[25] Jaitin DA (2006). Inquiring into the differential action of interferons (IFNs): an IFN-alpha2 mutant with enhanced affinity to IFNAR1 is functionally similar to IFN-beta. Mol Cell Biol.

[26] Kalie E (2008). The stability of the ternary interferon-receptor complex rather than the affinity to the individual subunits dictates differential biological activities. J Biol Chem.

[27] Damdinsuren B (2007). Stronger growth-inhibitory effect of interferon (IFN)-beta compared to IFN-alpha is mediated by IFN signaling pathway in hepatocellular carcinoma cells. Int J Oncol.

[28] Musella M (2021). The Yin and Yang of Type I IFNs in cancer promotion and immune activation. Biology.

[29] Parker BS, Rautela J, Hertzog PJ (2016). Antitumour actions of interferons: implications for cancer therapy. Nat Rev Cancer.

[30] Cheon H (2023). How cancer cells make and respond to interferon-I. Trends Cancer.

[31] Brzostek-Racine S (2011). The DNA damage response induces IFN. J Immunol.

[32] Mackenzie KJ (2017). cGAS surveillance of micronuclei links genome instability to innate immunity. Nature.

[33] Hartlova A (2015). DNA damage primes the type I interferon system via the cytosolic DNA sensor STING to promote anti-microbial innate immunity. Immunity.

[34] Black JRM, McGranahan N (2021). Genetic and non-genetic clonal diversity in cancer evolution. Nat Rev Cancer.

[35] Vendramin R, Litchfield K, Swanton C (2021). Cancer evolution: Darwin and beyond. EMBO J.

[36] Zhu X (2021). Cancer evolution: a means by which tumors evade treatment. Biomed Pharmacother.

[37] Zheng S, Asnani M, Thomas-Tikhonenko A (2019). Escape From ALL-CARTaz: leukemia immunoediting in the age of chimeric antigen receptors. Cancer J.

[38] Singal AG, Lampertico P, Nahon P (2020). Epidemiology and surveillance for hepatocellular carcinoma: new trends. J Hepatol.

[39] Wieland S (2014). Simultaneous detection of hepatitis C virus and interferon stimulated gene expression in infected human liver. Hepatology.

[40] Bigger CB, Brasky KM, Lanford RE (2001). DNA microarray analysis of chimpanzee liver during acute resolving hepatitis C virus infection. J Virol.

[41] Sarasin-Filipowicz M (2008). Interferon signaling and treatment outcome in chronic hepatitis C. Proc Natl Acad Sci USA.

[42] Yang YM, Kim SY, Seki E (2019). Inflammation and liver cancer: molecular mechanisms and therapeutic targets. Semin Liver Dis.

[43] Grivennikov SI, Greten FR, Karin M (2010). Immunity, inflammation, and cancer. Cell.

[44] von Locquenghien M, Rozalen C, Celia-Terrassa T (2021). Interferons in cancer immunoediting: sculpting metastasis and immunotherapy response. J Clin Invest.

[45] Noguchi M, Hirohashi S (1996). Cell lines from non-neoplastic liver and hepatocellular carcinoma tissue from a single patient. In Vitro Cell Dev Biol Anim.

[46] Burkart SS (2022). High resolution kinetic characterization and dynamic mathematical modeling of the RIG-I signaling pathway and the antiviral responses. Life Sci Alliance.

[47] Dao Thi VL (2020). Stem cell-derived polarized hepatocytes. Nat Commun.

[48] Pfaffl MW (2001). A new mathematical model for relative quantification in real-time RT-PCR. Nucleic Acids Res.

[49] Stirling DR (2021). Cell Profiler 4: improvements in speed, utility and usability. BMC Bioinformatics.

[50] Hoffmann M (2010). Fusion-active glycoprotein G mediates the cytotoxicity of vesicular stomatitis virus M mutants lacking host shut-off activity. J Gen Virol.

[51] Muller T (2020). Automated sample preparation with SP3 for low-input clinical proteomics. Mol Syst Biol.

[52] Vizcaino JA (2013). The PRoteomics IDEntifications (PRIDE) database and associated tools: status in 2013. Nucleic Acids Res.

[53] Heming S (2022). MSPypeline: a python package for streamlined data analysis of mass spectrometry-based proteomics. Bioinform Adv.

[54] Ritchie ME (2015). limma powers differential expression analyses for RNA-sequencing and microarray studies. Nucleic Acids Res.

[55] Maier BD (2022). Stochastic dynamics of Type-I interferon responses. PLoS Comput Biol.

[56] Johns TG (1992). Antiproliferative potencies of interferons on melanoma cell lines and xenografts: higher efficacy of interferon beta. J Natl Cancer Inst.

[57] van Koetsveld PM (2006). Potent inhibitory effects of type I interferons on human adrenocortical carcinoma cell growth. J Clin Endocrinol Metab.

[58] Lopez-Terrada D (2009). Hep G2 is a hepatoblastoma-derived cell line. Hum Pathol.

[59] Ishii T (2020). Analysis of HBV genomes integrated into the genomes of human hepatoma PLC/PRF/5 cells by HBV sequence capture-based next-generation sequencing. Genes.

[60] Hsu IC (1993). p53 gene mutation and integrated hepatitis B viral DNA sequences in human liver cancer cell lines. Carcinogenesis.

[61] Alexander JJ (1976). Establishment of a continuously growing cell line from primary carcinoma of the liver. S Afr Med J.

[62] Huh N, Utakoji T (1981). Production of HBs-antigen by two new human hepatoma cell lines and its enhancement by dexamethasone. Gan.

[63] Fukuyama K (2021). Gene expression profiles of liver cancer cell lines reveal two hepatocyte-like and fibroblast-like clusters. PLoS ONE.

[64] Lee JS, Thorgeirsson SS (2002). Functional and genomic implications of global gene expression profiles in cell lines from human hepatocellular cancer. Hepatology.

[65] Doi I (1976). Establishment of a cell line and its clonal sublines from a patient with hepatoblastoma. Gan.

[66] Nakabayashi H (1982). Growth of human hepatoma cells lines with differentiated functions in chemically defined medium. Cancer Res.

[67] Yuzugullu H (2009). Canonical Wnt signaling is antagonized by noncanonical Wnt5a in hepatocellular carcinoma cells. Mol Cancer.

[68] Nwosu ZC (2018). Liver cancer cell lines distinctly mimic the metabolic gene expression pattern of the corresponding human tumours. J Exp Clin Cancer Res.

[69] Aden DP (1979). Controlled synthesis of HBsAg in a differentiated human liver carcinoma-derived cell line. Nature.

[70] Doi I, Namba M, Sato J (1975). Establishment and some biological characteristics of human hepatoma cell lines. Gann.

[71] Park JG (1995). Characterization of cell lines established from human hepatocellular carcinoma. Int J Cancer.

[72] Fuchs BC (2008). Epithelial-to-mesenchymal transition and integrin-linked kinase mediate sensitivity to epidermal growth factor receptor inhibition in human hepatoma cells. Cancer Res.

[73] Edmondson HA, Steiner PE (1954). Primary carcinoma of the liver-a study of 100 cases among 48,900 necropsies. Cancer.

[74] Definition of malignancy - National Cancer Institute. 2022 23.05.2022]; Definition of malignancy - National Cancer Institute]. https://www.cancer.gov/publications/dictionaries/cancer-terms/def/malignancy.

[75] Wu X (2018). Pan-genotype hepatitis E virus replication in stem cell-derived hepatocellular systems. Gastroenterology.

[76] Steinbichler TB (2020). Cancer stem cells and their unique role in metastatic spread. Semin Cancer Biol.

[77] Deryugina EI, Quigley JP (2006). Matrix metalloproteinases and tumor metastasis. Cancer Metastasis Rev.

[78] Kano A (1999). IRF-1 is an essential mediator in IFN-gamma-induced cell cycle arrest and apoptosis of primary cultured hepatocytes. Biochem Biophys Res Commun.

[79] Kok F (2020). Disentangling molecular mechanisms regulating sensitization of interferon alpha signal transduction. Mol Syst Biol.

[80] Levin D (2014). Multifaceted activities of type I interferon are revealed by a receptor antagonist. Sci Signal.

[81] Francois-Newton V (2011). USP18-based negative feedback control is induced by type I and type III interferons and specifically inactivates interferon alpha response. PLoS ONE.

[82] Tsai MH, Pai LM, Lee CK (2019). Fine-tuning of type I interferon response by STAT3. Front Immunol.

[83] Borden EC (2019). Interferons alpha and beta in cancer: therapeutic opportunities from new insights. Nat Rev Drug Discov.

[84] Rautela J (2015). Loss of host type-I IFN signaling accelerates metastasis and impairs NK-cell antitumor function in multiple models of breast cancer. Cancer Immunol Res.

[85] Brunda MJ, Rosenbaum D, Stern L (1984). Inhibition of experimentally-induced murine metastases by recombinant alpha interferon: correlation between the modulatory effect of interferon treatment on natural killer cell activity and inhibition of metastases. Int J Cancer.

[86] Nishimura J (1985). Antitumor and antimetastatic activities of human recombinant interferon alpha A/D. Clin Exp Metastasis.

[87] Ortiz A, Fuchs SY (2017). Anti-metastatic functions of type 1 interferons: Foundation for the adjuvant therapy of cancer. Cytokine.

[88] Sistigu A (2014). Cancer cell-autonomous contribution of type I interferon signaling to the efficacy of chemotherapy. Nat Med.

[89] Cheon H (2021). PD-L1 sustains chronic, cancer cell-intrinsic responses to type I interferon, enhancing resistance to DNA damage. Proc Natl Acad Sci U S A.

[90] Boelens MC (2014). Exosome transfer from stromal to breast cancer cells regulates therapy resistance pathways. Cell.

[91] Yang CH (2000). IFNalpha/beta promotes cell survival by activating NF-kappa B. Proc Natl Acad Sci USA.

[92] Zhao H (2015). The roles of interferon-inducible p200 family members IFI16 and p204 in innate immune responses, cell differentiation and proliferation. Genes Dis.

[93] Honkala AT, Tailor D, Malhotra SV (2019). Guanylate-Binding Protein 1: An Emerging Target in Inflammation and Cancer. Front Immunol.

[94] Binder P (2021). Optimal ligand discrimination by asymmetric dimerization and turnover of interferon receptors. Proc Natl Acad Sci USA.

[95] Oyler-Yaniv A (2017). A tunable diffusion-consumption mechanism of cytokine propagation enables plasticity in cell-to-cell communication in the immune system. Immunity.

[96] Yano H (2006). Growth inhibitory effects of pegylated IFN alpha-2b on human liver cancer cells in vitro and in vivo. Liver Int.

[97] Sayeed A (2020). Profiling the circulating mRNA transcriptome in human liver disease. Oncotarget.

[98] Choi HY (2020). p53 destabilizing protein skews asymmetric division and enhances NOTCH activation to direct self-renewal of TICs. Nat Commun.

[99] Pipas JM (2009). SV40: cell transformation and tumorigenesis. Virology.

[100] Muller PA, Vousden KH, Norman JC (2011). p53 and its mutants in tumor cell migration and invasion. J Cell Biol.

[101] Xia M, Land H (2007). Tumor suppressor p53 restricts Ras stimulation of RhoA and cancer cell motility. Nat Struct Mol Biol.

[102] Damdinsuren B (2007). Interferon alpha receptors are important for antiproliferative effect of interferon-alpha against human hepatocellular carcinoma cells. Hepatol Res.

[103] Zhang KX (2010). Down-regulation of type I interferon receptor sensitizes bladder cancer cells to vesicular stomatitis virus-induced cell death. Int J Cancer.

[104] Sabbatino F (2016). Antitumor activity of BRAF inhibitor and IFNalpha combination in BRAF-mutant melanoma. J Natl Cancer Inst.

[105] Wang JW (2018). Decreased methylation of IFNAR gene promoter from peripheral blood mononuclear cells is associated with oxidative stress in chronic hepatitis B. J Interferon Cytokine Res.

[106] Conway KE (2000). TMS1, a novel proapoptotic caspase recruitment domain protein, is a target of methylation-induced gene silencing in human breast cancers. Cancer Res.

[107] Schreiber G, Piehler J (2015). The molecular basis for functional plasticity in type I interferon signaling. Trends Immunol.

[108] Levin D, Harari D, Schreiber G (2011). Stochastic receptor expression determines cell fate upon interferon treatment. Mol Cell Biol.

[109] Moraga I (2009). Receptor density is key to the alpha2/beta interferon differential activities. Mol Cell Biol.

[110] Wagner TC (2004). Interferon receptor expression regulates the antiproliferative effects of interferons on cancer cells and solid tumors. Int J Cancer.

[111] Pidugu VK (2019). Emerging functions of human IFIT proteins in cancer. Front Mol Biosci.

[112] Kirchhoff S, Schaper F, Hauser H (1993). Interferon regulatory factor 1 (IRF-1) mediates cell growth inhibition by transactivation of downstream target genes. Nucleic Acids Res.

[113] Tanaka N (1994). Cellular commitment to oncogene-induced transformation or apoptosis is dependent on the transcription factor IRF-1. Cell.

[114] Honke N (2016). Multiple functions of USP18. Cell Death Dis.

[115] Vuillier F (2019). USP18 and ISG15 coordinately impact on SKP2 and cell cycle progression. Sci Rep.

[116] Mustachio LM (2018). Evidence for the ISG15-Specific Deubiquitinase USP18 as an antineoplastic target. Cancer Res.

[117] Arimoto KI (2017). STAT2 is an essential adaptor in USP18-mediated suppression of type I interferon signaling. Nat Struct Mol Biol.

[118] Malakhova OA (2006). UBP43 is a novel regulator of interferon signaling independent of its ISG15 isopeptidase activity. EMBO J.

[119] Francois-Newton V (2012). USP18 establishes the transcriptional and anti-proliferative interferon alpha/beta differential. Biochem J.

