# Supplementary material for: Tumour cells can escape antiproliferative pressure by interferon-β through immunoediting of interferon receptor expression
Source: Cancer Cell Int. 2023 Dec 8;23:315. doi: 10.1186/s12935-023-03150-y (PMC10709914; doi:10.1186/s12935-023-03150-y)
Supplement: Supplementary file 1 — Additional file 1: Figure S1: Fluorescence microscopy images during selection experiment. 5% or 10% (as indicated) of IFN-β unresponsive IFNAR DKO A549 cells (nuclear H2B-mCherry, magenta) were cocultivated with control A549NT cells (cytoplasmatic GFP, cyan). IFNAR1 KO gave a relative advantage to resistant cells in conditions continuously stimulated with 5000 IU/ml IFN-β over mock. [file 12935_2023_3150_MOESM1_ESM.pdf]

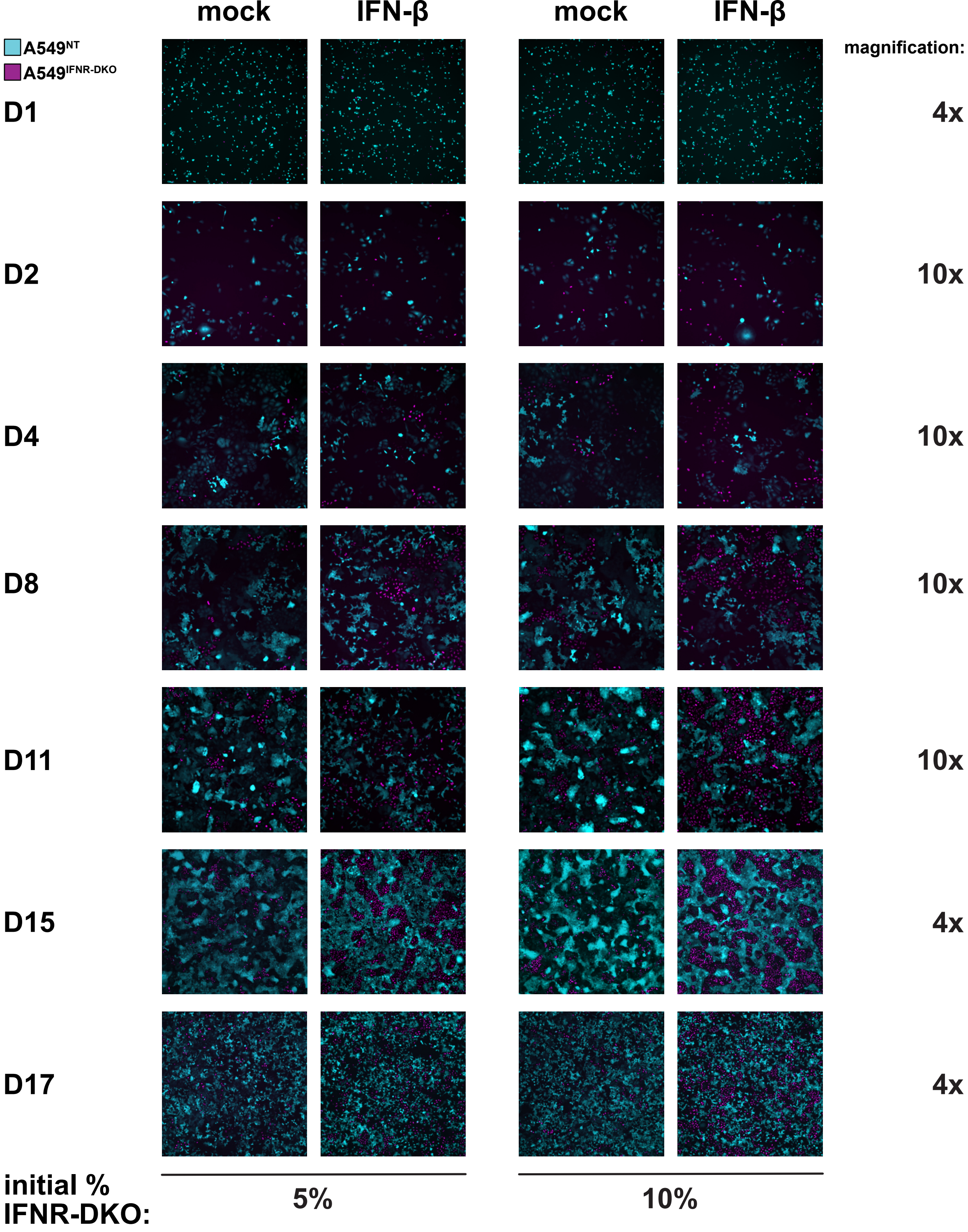

mock
IFN-β
mock
IFN-β
A549NT
A549IFNR-DKO
magnification:
D1
4x
D2
10x
D4
10x
D8
10x
D11
10x
D15
4x
D17
4x
initial % IFNR-DKO:
5%
10%
