# Supplementary figures and images for: Tumour cells can escape antiproliferative pressure by interferon-β through immunoediting of interferon receptor expression
Source: Cancer Cell Int. 2023 Dec 8;23:315. doi: 10.1186/s12935-023-03150-y (PMC10709914; doi:10.1186/s12935-023-03150-y)

# Interim Analysis of Growth Rate

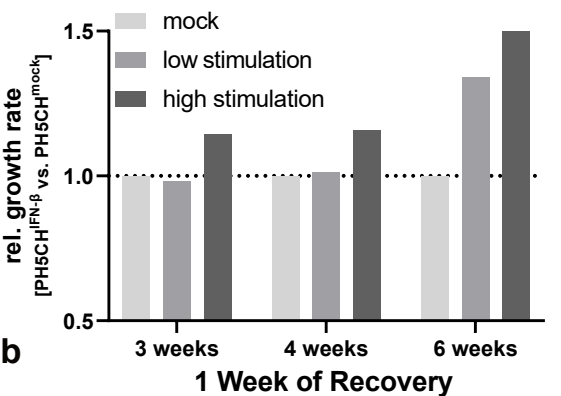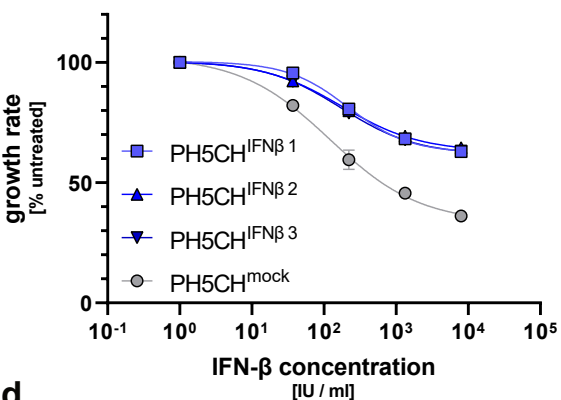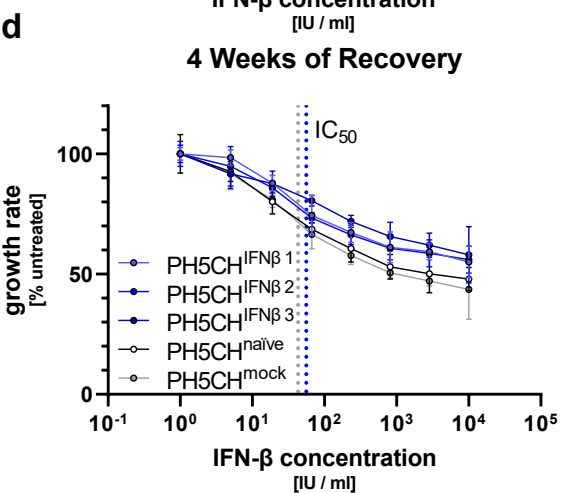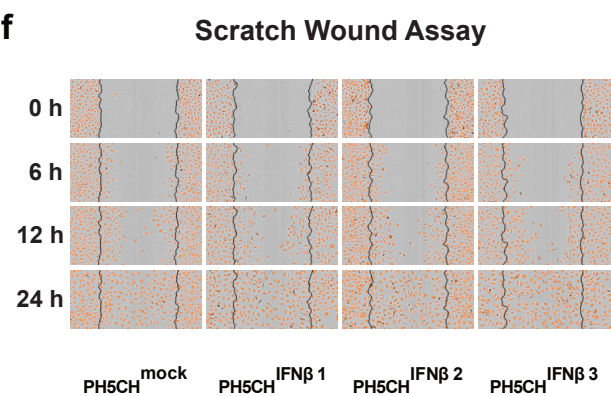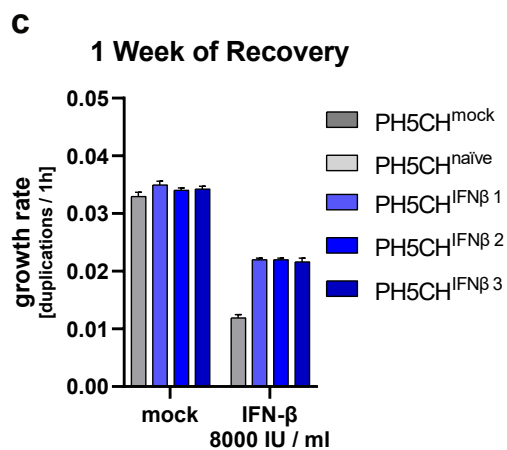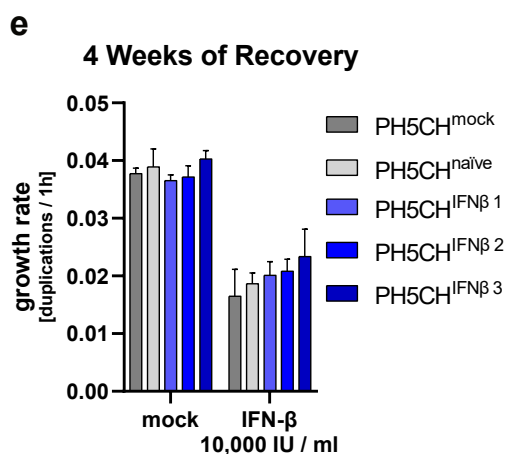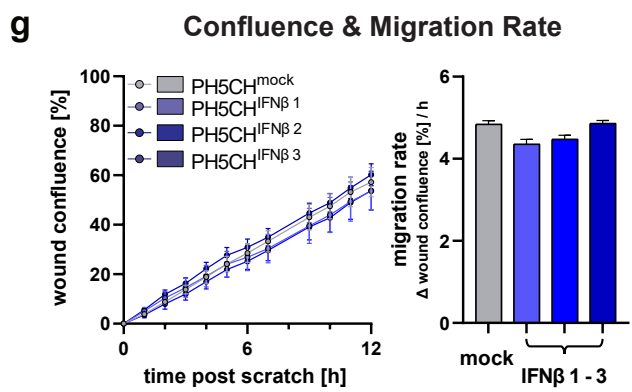

Supplement: Supplementary file 2 — Additional file 2: Figure S2: Comparison of distinct populations of PH5CH that were selected by continuous IFN-β pressure. a Interim analysis of IFN-β-inhibited cell growth after 3, 4 or 6 weeks of selection and one week of recovery. Relative growth of PH5CHIFN−β vs. PH5CHmock upon stimulation with low (100–222 IU/ml) or high dose (1000–1333 IU/ml) of IFN-β. b-e Three separate populations of PH5CH (PH5CHIFN−β 1–3) were selected by continuous IFN-β pressure (2000 IU/ml) over six weeks (see Fig. 2). Growth rates determined after one and four weeks of recovery and compared to a population passaged in parallel in absence of IFN-β (PH5CHmock) and/or a freshly thawed population of PH5CH (PH5CHnaïve). b + d Growth rates relative to mock at different IFN-β concentrations. Untreated condition is represented at x = 100 IU/ml for simplicity. PH5CHnaïve were thawed 1 week before the experiment and behaved very comparable to PH5CHmock, whereas PH5CHIFN−β populations were less impaired in growth by IFN-β. c + e Absolute growth rates in mock condition vs. stimulation with 8000 IU/ml IFN-β. f + g Migration rate of PH5CHmock and PH5CHIFN−β 1–3 determined in scratch wound assay. f Representative microscopic images. Wound margins at 0 h are demarcated in black, nuclei in orange. g Migration rate calculated as in Fig. 3 during period of highest motility (0–12 h). Graphs display mean ± SD. [file 12935_2023_3150_MOESM2_ESM.pdf]

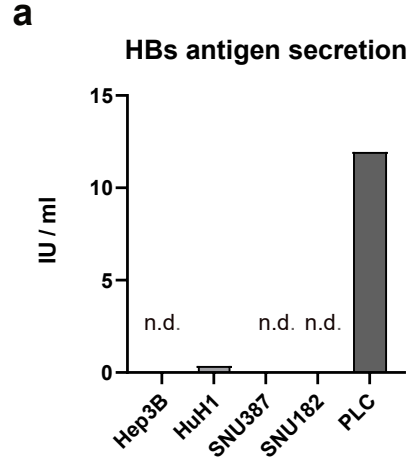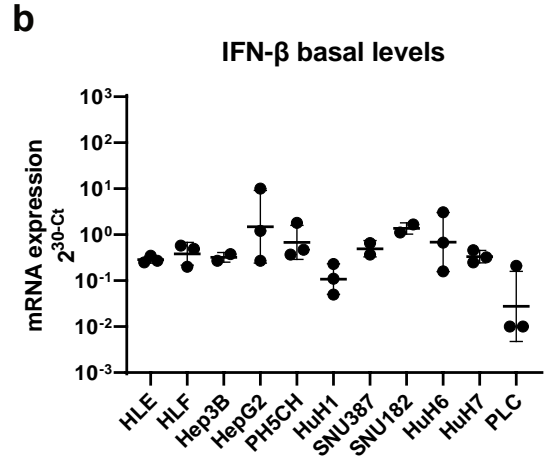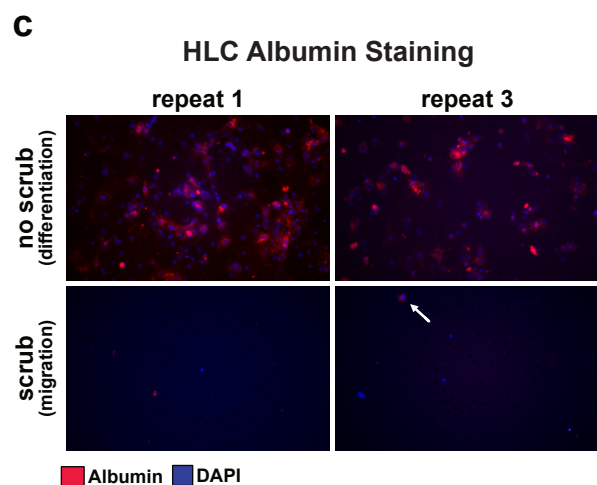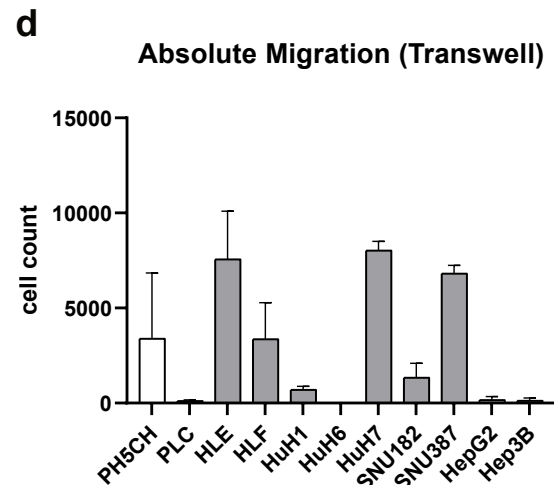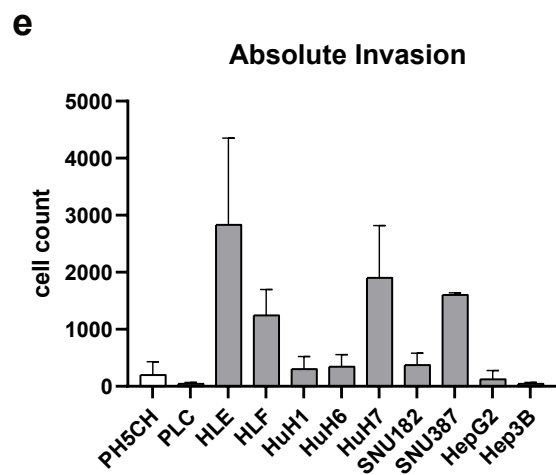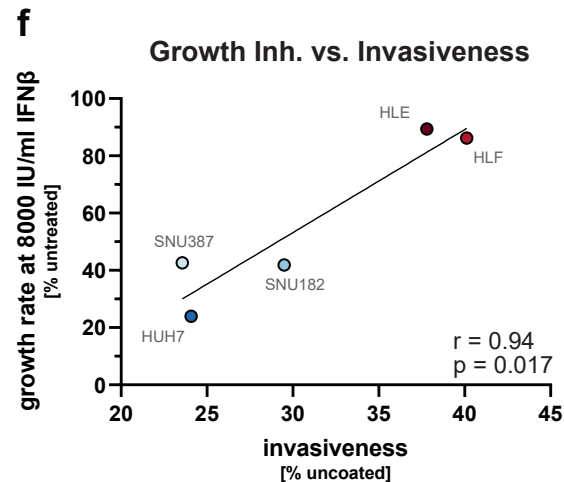

Supplement: Supplementary file 3 — Additional file 3: Figure S3: Assessment of immune status and malignity of ten liver cancer cell lines.. a Levels of secreted HBs antigen in the supernatant of five hepatoma cell lines from our panel that have been reported to harbour integrates of HBV were measured using The ARCHITECT HBsAg assay. b Expression of IFN-β at the basal level shown as absolute mRNA levels. The graph displays mean (± SD) of three separate replicate wells, except for the Hep3B, SNU182 and SNU387 cell lines, as indicated. Note all cell lines showed signals around CT = 30 being close to the detection limit. No IFN-β protein was detected in supernatants by ELISA. c HLC on uncoated membranes 24 h after seeding. No scrub (upper images) visualises all HLC cells. Hoechst and albumin staining show differentiation into HLC. After scrubbing (lower images), only cells that migrated to the lower chamber remain. The arrow points out a single albumin expressing cell on the lower surface, while other migrated cells were albumin negative (not hepatocyte-like). Representative images (20 × magnification). d + e: Total counts of cells that migrated through uncoated (d) respectively matrigel-coated (d) transwell membranes after 24 h. Fast migrating PH5CH, HLE, HLF and SNU387 were seeded at 2.5 × 10^4^, other cell lines at 1 × 10^5^ cells/chamber. f Relative growth rates at 8000 IU/ml IFN-β plotted against respective invasiveness of cell lines showing > 1000 migrated cells on uncoated membrane as determined in Fig. 3. Linear regression was performed and Pearson correlation was calculated. [file 12935_2023_3150_MOESM3_ESM.pdf]

HLE

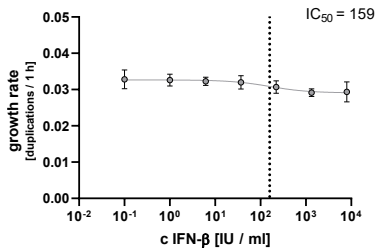

HLF

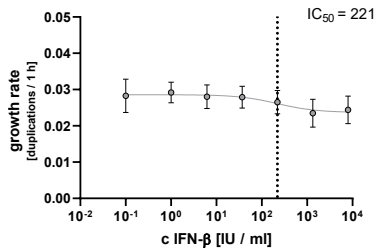

Hep3B

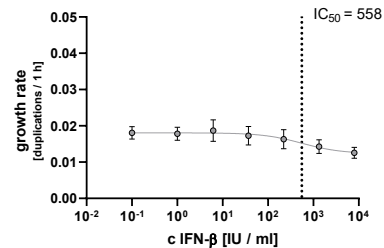

HepG2

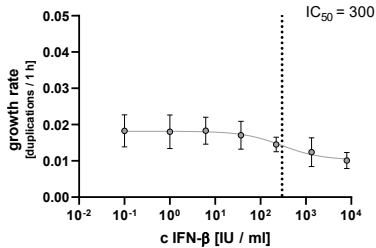

PH5CH

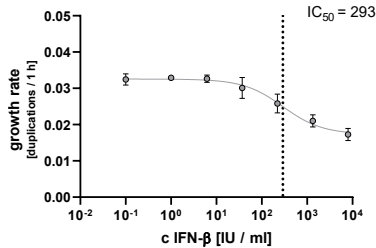

HuH1

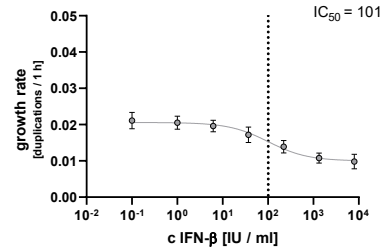

SNU387

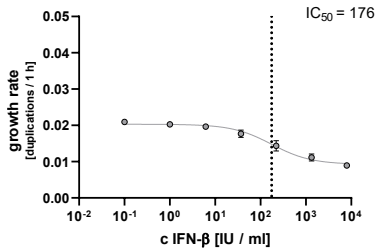

SNU182

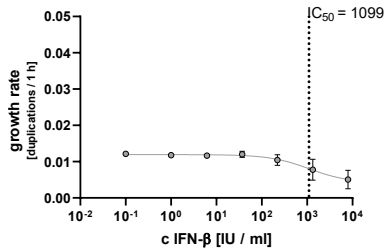

HuH6

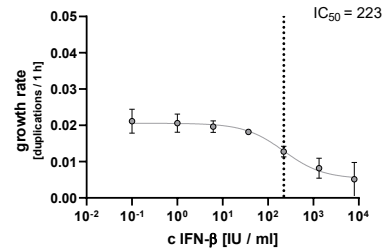

HuH7

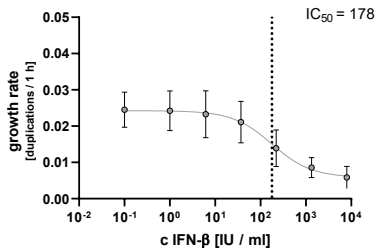

PLC/PRF/5

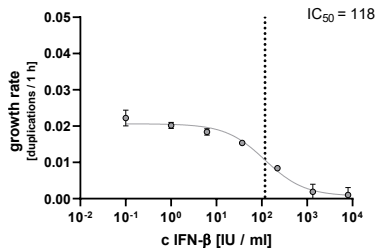

Supplement: Supplementary file 4 — Additional file 4: Figure S4: Growth inhibition of hepatoma cells by increasing concentrations of IFN-β. Mock stimulation is represented at x = 10–1 IU/ml for simplicity. IC50 included as orientation (dashed vertical line). Graphs display mean ± SD of three independently repeated experiments, each consisting of 12 technical replicates (4 images from 3 separate wells per condition). Determination of growth rates as described in methods. [file 12935_2023_3150_MOESM4_ESM.pdf]

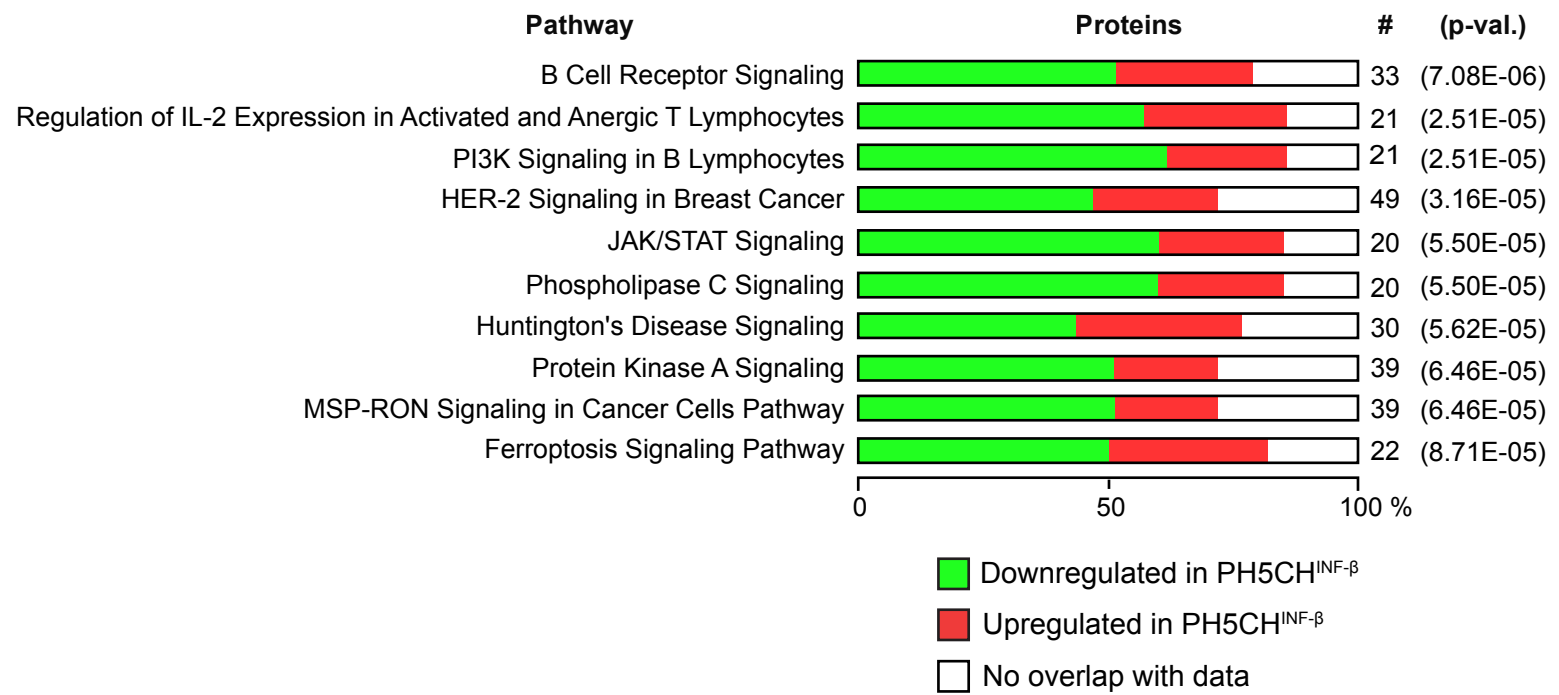

Supplement: Supplementary file 6 — Additional file 6: Figure S6: Long-term selected PH5CH cells were subjected to full-proteome mass spectrometry analysis and differentially expressed proteins (PH5CHIFN−β versus PH5CHmock) were analysed by IPA pathway analysis (Qiagen). For full IPA analysis report, see Additional file 7. [file 12935_2023_3150_MOESM6_ESM.pdf]
