# Supplementary material for: Tumour cells can escape antiproliferative pressure by interferon-β through immunoediting of interferon receptor expression
Source: Cancer Cell Int. 2023 Dec 8;23:315. doi: 10.1186/s12935-023-03150-y (PMC10709914; doi:10.1186/s12935-023-03150-y)
Supplement: Supplementary file 5 — Additional file 5: Figure S5: Hepatoma cell lines have an intact response to viral infection and the differential antiproliferative effect of IFN-β negatively correlates with the expression of baseline levels of IFNAR2. a + b Hepatoma cells (3.5 × 10^5^) were mock treated or infected with VSV-MQ recombinant virus expressing eGFP at an MOI of 10. After 24 h, cells were analysed via qRT-PCR for their permissiveness to viral replication (a) and mounting of an antiviral response (b). c + d Expression levels of IFNAR1 and IFNAR2 subunits quantified independently of the housekeeping genes. e + f Relative growth rates at 8000 IU/ml IFN-β plotted against IFNAR1 (e) or IFNAR2 (f) expression. Pearson correlation and linear regression was calculated. All graphs display mean (± SD) of three separate replicate wells, except for Hep3B, SNU182 and SNU387 cell lines, as indicated. [file 12935_2023_3150_MOESM5_ESM.pdf]

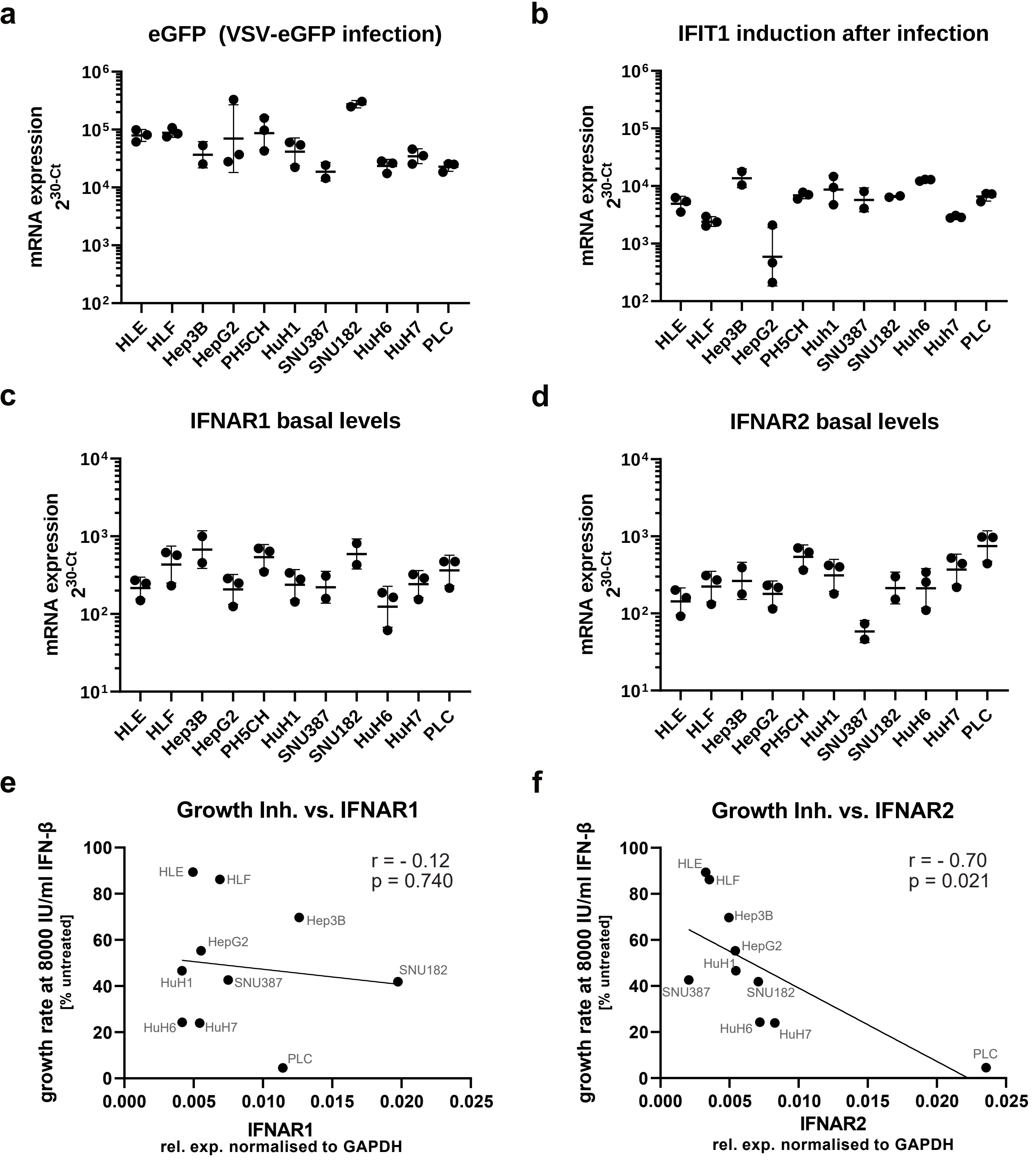
a
eGFP (VSV-eGFP infection)
mRNA expression
2^30-Ct
HLE
HLF
Hep3B
HepG2
PH5CH
HuH1
SNU387
SNU182
HuH6
HuH7
PLC
b
IFIT1 induction after infection
mRNA expression
2^30-Ct
HLE
HLF
Hep3B
HepG2
PH5CH
Huh1
SNU387
SNU182
Huh6
Huh7
PLC
c
IFNAR1 basal levels
mRNA expression
2^30-Ct
d
IFNAR2 basal levels
mRNA expression
2^30-Ct
e
Growth Inh. vs. IFNAR1
growth rate at 8000 IU/ml IFN-β
[% untreated]
r = - 0.12
p = 0.740
HLE
HLF
Hep3B
HepG2
HuH1
SNU387
SNU182
HuH6
HuH7
PLC
IFNAR1
rel. exp. normalised to GAPDH
f
Growth Inh. vs. IFNAR2
growth rate at 8000 IU/ml IFN-β
[% untreated]
r = - 0.70
p = 0.021
IFNAR2
rel. exp. normalised to GAPDH
