# Supplementary material for: Tumour cells can escape antiproliferative pressure by interferon-β through immunoediting of interferon receptor expression
Source: Cancer Cell Int. 2023 Dec 8;23:315. doi: 10.1186/s12935-023-03150-y (PMC10709914; doi:10.1186/s12935-023-03150-y)
Supplement: Supplementary file 7 — Additional file 7. IPA analysis report.pdf. This file contains the full report of the Qiagen IPA analysis of the proteomes shown in Additional file 6: Fig. S6 [file 12935_2023_3150_MOESM7_ESM.pdf]

Analysis Name: PH5CH-LTm_vs_PH5CM-LTb1 - 2023-10-06 02:46 pm
Analysis Creation Date: 2023-10-06
Build version: exported
Content version: 94302991 (Release Date: 2023-05-27)

Experiment Metadata

Name	Value

Analysis Settings

Reference set: Ingenuity Knowledge Base (Genes Only)
Relationship to include: Direct and Indirect
Includes Endogenous Chemicals
Optional Analyses: My Pathways My List

Filter Summary:
Consider only molecules and/or relationships where
(species = Human) AND
(confidence = Experimentally Observed) AND
(cell lines = Hep3B OR Hepatoma Cell Lines not otherwise specified OR HuH7 OR Other Hepatoma Cell Lines OR Cell Line not otherwise specified OR HepG2) AND
(mol. types = biologic drug OR canonical pathway OR chemical - endogenous mammalian OR chemical - endogenous non-mammalian OR

(c) 2000-2023 QIAGEN. All rights reserved.

chemical - kinase inhibitor OR chemical - other OR chemical - protease inhibitor OR chemical drug OR chemical reagent OR chemical toxicant OR complex OR cytokine OR disease OR enzyme OR function OR fusion gene/product OR G-protein coupled receptor OR group OR growth factor OR ion channel OR kinase OR ligand-dependent nuclear receptor OR mature microRNA OR microRNA OR other OR peptidase OR phosphatase OR transcription regulator OR translation regulator OR transmembrane receptor OR transporter) AND
(data sources = An Open Access Database of Genome-wide Association Results OR BIND OR BioGRID OR Catalogue Of Somatic Mutations In Cancer (COSMIC) OR Chemical Carcinogenesis Research Information System (CCRIS) OR Clinical Genome Resource (ClinGen) OR ClinicalTrials.gov OR ClinVar OR Cognia OR DIP OR DrugBank OR Gene Ontology (GO) OR GVK Biosciences OR Hazardous Substances Data Bank (HSDB) OR HumanCyc OR Ingenuity Expert Findings OR Ingenuity ExpertAssist Findings OR IntAct OR Interactome studies OR MIPS OR miRBase OR miRecords OR Mouse Genome Database (MGD) OR Obesity Gene Map Database OR Online Mendelian Inheritance in Man (OMIM) OR Reactome OR TarBase OR TargetScan Human OR TargetScan Mouse)

Top Canonical Pathways

Name	p-value	Overlap
**B Cell Receptor Signaling**	7.06E-06	78.8 % 26/33
**Regulation of IL-2 Expression in Activated and Anergic T Lymphocytes**	2.52E-05	85.7 % 18/21
**PI3K Signaling in B Lymphocytes**	2.52E-05	85.7 % 18/21
**HER-2 Signaling in Breast Cancer**	3.15E-05	69.4 % 34/49
**Phospholipase C Signaling**	5.47E-05	85.0 % 17/20

Top Upstream Regulators

Upstream Regulators

(c) 2000-2023 QIAGEN. All rights reserved.

Name	p-value	Predicted Activation
**SP1**	1.96E-03	
**SREBF1**	2.30E-03	
**NQO1**	2.79E-02	
**PTPN6**	2.79E-02	

Causal Network

Name	p-value	Predicted Activation
**NQO1**	2.79E-02	
**PTPN6**	2.79E-02	
**TGFB1**	1.05E-01	
**MYC**	1.05E-01	
**TGFB1**	1.10E-01	

Top Diseases and Bio Functions

Diseases and Disorders

Name	p-value range	# Molecules
**Infectious Diseases**	4.45E-02 - 1.47E-02	24
**Organismal Injury and Abnormalities**	4.45E-02 - 1.47E-02	24
**Gastrointestinal Disease**	4.45E-02 - 4.45E-02	5

(c) 2000-2023 QIAGEN. All rights reserved.

INGENUITY PATHWAY ANALYSIS

**Hepatic System Disease**	4.45E-02 - 4.45E-02	5
**Inflammatory Disease**	4.45E-02 - 4.45E-02	5

Molecular and Cellular Functions

Name	p-value range	# Molecules
**Lipid Metabolism**	2.76E-02 - 2.76E-02	4
**Molecular Transport**	2.76E-02 - 2.76E-02	4
**Nucleic Acid Metabolism**	2.76E-02 - 2.76E-02	4
**Small Molecule Biochemistry**	4.45E-02 - 2.76E-02	8
**Carbohydrate Metabolism**	4.45E-02 - 4.45E-02	5

Physiological System Development and Function

Name	p-value range	# Molecules
**Digestive System Development and Function**	4.45E-02 - 4.45E-02	5
**Hepatic System Development and Function**	4.45E-02 - 4.45E-02	5
**Organ Development**	4.45E-02 - 4.45E-02	5

Top Tox Functions

Assays: Clinical Chemistry and Hematology

(c) 2000-2023 QIAGEN. All rights reserved.

INGENUITY PATHWAY ANALYSIS

Name	p-value range	# Molecules
**Decreased Levels of Albumin**	4.08E-01 - 4.08E-01	1

Hepatotoxicity

Name	p-value range	# Molecules
**Liver Inflammation/Hepatitis**	1.00E00 - 4.45E-02	6
**Hepatocellular carcinoma**	4.08E-01 - 4.08E-01	1
**Liver Hyperplasia/Hyperproliferation**	4.08E-01 - 4.08E-01	1

Top Regulator Effect Networks

Top Networks

ID	Associated Network Functions	Score
**1**	Cellular Development, Cellular Growth and Proliferation, Cardiovascular System Development and Function	13
**2**	Carbohydrate Metabolism, Lipid Metabolism, Molecular Transport	8

(c) 2000-2023 QIAGEN. All rights reserved.

**3**	Connective Tissue Development and Function, Tissue Morphology, Cell Death and Survival	7
**4**	Cell Death and Survival, Organismal Injury and Abnormalities, Cancer	4
**5**	Cell Cycle, Gene Expression, Cellular Movement	3

Top Tox Lists

Name	p-value	Overlap
**PPAR/RXR Activation**	9.04E-04	64.4 % 29/45
**Mitochondrial Dysfunction**	9.88E-03	61.8 % 21/34
**Renal Necrosis/Cell Death**	1.15E-02	50.4 % 65/129
**NRF2-mediated Oxidative Stress Response**	1.47E-02	58.5 % 24/41
**Cell Cycle: G2/M DNA Damage Checkpoint Regulation**	1.72E-02	75.0 % 9/12

Top My Lists

Top My Pathways

Top ML Disease Pathways

(c) 2000-2023 QIAGEN. All rights reserved.

Name	p-value	Overlap
**Hamartomatous polyposis syndrome**	4.83E-05	100.0 % 11/11
**Esophagus tumor**	3.66E-04	91.7 % 11/12
**Fibroma**	1.83E-03	100.0 % 7/7
**Chronic myelomonocytic leukemia**	1.83E-03	100.0 % 7/7
**Myelomonocytic leukemia**	1.83E-03	100.0 % 7/7

Top Analysis-Ready Molecules

Expr Log Ratio

Molecules	Expr. Value	Chart
**KRT10**	↑ 2.541	
**FOXO1**	↑ 1.747	
**TNFRSF10A**	↑ 1.694	
**ADH1C**	↑ 1.631	
**VLDLR**	↑ 1.286	
**APOA1**	↑ 1.166	
**TFPI**	↑ 0.945	
**STING1**	↑ 0.934	
**TF**	↑ 0.862	
**KRT18**	↑ 0.839	

Expr Log Ratio

(c) 2000-2023 QIAGEN. All rights reserved.

Molecules	Expr. Value	Chart
**VCAN**	↓ -2.244	
**PYCARD**	↓ -1.882	
**AKIRIN2**	↓ -1.631	
**BACH1**	↓ -1.529	
**TIMP1**	↓ -1.295	
**NT5E**	↓ -1.241	
**CES1**	↓ -1.186	
**EPCAM**	↓ -1.184	
**CD44**	↓ -1.180	
**WNT5B**	↓ -1.113	

(c) 2000-2023 QIAGEN. All rights reserved.

INGENUITY PATHWAY ANALYSIS
